# Behavioural components and delivery features of early childhood obesity prevention interventions: intervention coding of studies in the TOPCHILD Collaboration systematic review

**DOI:** 10.1186/s12966-025-01708-9

**Published:** 2025-02-05

**Authors:** Brittany J. Johnson, Paul M. Chadwick, Samantha Pryde, Anna Lene Seidler, Kylie E. Hunter, Mason Aberoumand, Jonathan G. Williams, Hei In Lau, Sol Libesman, Jannik Aagerup, Angie Barba, Louise A. Baur, Samantha Morgillo, Lee Sanders, Sarah Taki, Kylie D. Hesketh, Karen Campbell, Alexandra Manson, Alison Hayes, Angela Webster, Charles Wood, Denise A. O’Connor, Karen Matvienko-Sikar, Kristy Robledo, Lisa Askie, Luke Wolfenden, Rachael Taylor, H. Shonna Yin, Vicki Brown, Alexander Fiks, Alison Ventura, Ata Ghaderi, Barry J. Taylor, Cathleen Stough, Christine Helle, Cristina Palacios, Eliana M. Perrin, Elizabeth Reifsnider, Finn Rasmussen, Ian M. Paul, Jennifer S. Savage, Jessica Thomson, Jinan Banna, Junilla Larsen, Kaumudi Joshipura, Ken K. Ong, Levie Karssen, Li Ming Wen, Márcia Vitolo, Margrethe Røed, Maria Bryant, Maribel Campos Rivera, Mary Jo Messito, Natalia Golova, Nina Cecilie Øverby, Rachel Gross, Rajalakshmi Lakshman, Rebecca Byrne, Russell L. Rothman, Sharleen O’Reilly, Stephanie Anzman-Frasca, Vera Verbestel, Claudio Maffeis, Kayla de la Haye, Sarah-Jeanne Salvy, Seema Mihrshahi, Janani Ramachandran, Paola Seffrin Baratto, Rebecca K. Golley, on behalf of the TOPCHILD Collaboration, Brittany J. Johnson, Paul M. Chadwick, Samantha Pryde, Anna Lene Seidler, Kylie E. Hunter, Mason Aberoumand, Jonathan G. Williams, Sol Libesman, Jannik Aagerup, Angie Barba, Louise A. Baur, Lee Sanders, Sarah Taki, Kylie D. Hesketh, Karen Campbell, Alison Hayes, Angela Webster, Charles Wood, Denise A. O’Connor, Karen Matvienko-Sikar, Kristy Robledo, Lisa Askie, Luke Wolfenden, Rachael Taylor, H. Shonna Yin, Vicki Brown, Alexander Fiks, Alison Ventura, Ata Ghaderi, Barry J. Taylor, Cathleen Stough, Christine Helle, Cristina Palacios, Eliana M. Perrin, Elizabeth Reifsnider, Finn Rasmussen, Ian M. Paul, Jennifer S. Savage, Jessica Thomson, Jinan Banna, Junilla Larsen, Kaumudi Joshipura, Levie Karssen, Li Ming Wen, Márcia Vitolo, Margrethe Røed, Maria Bryant, Mary Jo Messito, Natalia Golova, Nina Cecilie Øverby, Rachel Gross, Rajalakshmi Lakshman, Rebecca Byrne, Russell L. Rothman, Sharleen O’Reilly, Stephanie Anzman-Frasca, Claudio Maffeis, Kayla de la Haye, Sarah-Jeanne Salvy, Seema Mihrshahi, Rebecca K. Golley, Anne-Louise Heath, David McCormick, Katie Angotti, Kim Roberts, Julia Valmorbida, David Nguyen, Nipun Shrestha, Chris Rissel, David Espinoza, Ian Marschner, Lucinda Bell, Lukas Staub, Michelle Sue-See, Peter Godolphin, Wendy Smith, Alison Karasz, Amanda Thompson, Ana Maria Linares, Ana Perez Exposito, Carolina González Acero, Cindy-Lee Dennis, David McCormick, Deborah Jacobvitz, Elizabeth Widen, Emily Oken, Eric Hodges, Eva Corpeleijn, Heather Wasser, Hein Raat, Hongping Xia, Ken Ong, Lene Kierkegaard, Logan Manikam, Lynne Daniels, Maribel Campos Rivera, Michael Goran, Priyanka Patil, Pujitha Wickramasinghe, Tiffany Rybak, Trine Pedersen, Shannon Whaley, Vasana Kiridana, Vera Verbeste

**Affiliations:** 1 College of Nursing and Health Sciences, Flinders University, Caring Futures Institute, Adelaide, Australia; 2 Centre for Behaviour Change, University College London, London, UK; 3 National Health and Medical Research Council (NHMRC) Clinical Trials Centre, University of Sydney, Camperdown, Australia; 4 Sydney Medical School, The University of Sydney, Camperdown, Australia; 5 Pediatrics and Health Policy, Stanford University, Stanford, USA; 6 Population Health Research and Evaluation Hub, Sydney Local Health District, Camperdown, Australia; 7 School of Public Health, Faculty of Medicine and Health, The University of Sydney, Camperdown, Australia; 8 Institute for Physical Activity and Nutrition, Deakin University, Geelong, Australia; 9 Department of Pediatrics, Duke University School of Medicine, Durham, NC USA; 10 School of Public Health and Preventive Medicine, Monash University, Clayton, Australia; 11 School of Public Health, University College Cork, Cork, Ireland; 12 School of Medicine and Public Health, The University of Newcastle, Newcastle, Australia; 13 University of Otago, Dunedin, New Zealand; 14 Departments of Pediatrics and Population Health, NYU Grossman School of Medicine, New York, USA; 15 Deakin Health Economics, Institute for Health Transformation, Deakin University, Geelong, Australia; 16 Clinical Futures and Department of Pediatrics, Children’s Hospital of Philadelphia and Perelman School of Medicine at the University of Pennsylvania, Philadelphia, USA; 17 Department of Kinesiology and Public Health, Bailey College of Science and Math, California Polytechnic State University, San Luis Obispo, USA; 18 Department of Clinical Neuroscience, Division of Psychology, Karolinska Institutet, Solna, Sweden; 19 Department of Psychology, University of Cincinnati, Cincinnati, USA; 20 Department of Nutrition and Public Health, University of Agder, Kristiansand, Norway; 21 Department of Dietetics and Nutrition, Florida International University, Miami, USA; 22 Department of Pediatrics, School of Medicine and School of Nursing, Johns Hopkins University, Baltimore, USA; 23 Arizona State University, Tempe, USA; 24 Department of Global Public Health, Karolinska Institutet, Solna, Sweden; 25 Penn State College of Medicine, Hershey, USA; 26 The Center for Childhood Obesity Research, Department of Nutritional Sciences at The Pennsylvania State University, University Park, USA; 27 US Department of Agriculture, Agricultural Research Service, Maryland, USA; 28 University of Hawaii, Manoa, USA; 29 Behavioural Science Institute, Radboud University, Nijmegen, the Netherlands; 30 Harvard Chan School of Public Health, Ahmedabad University School of Public Health, Boston, USA; 31 Medical Research Centre Epidemiology Unit, Institute of Metabolic Science, University of Cambridge, Cambridge, UK; 32 Medical Sciences Campus, University of Puetro Rico, San Juan, Puerto Rico; 33 University of York, York, UK; 34 COHeAL University of Puerto Rico Medical Sciences Campus, San Juan, Puerto Rico; 35 New York University Grossman School of Medicine, New York, USA; 36 Hasbro Children’s Hospital, Warren Alpert School of Medicine of Brown University, Providence, USA; 37 Department of Pediatrics, Department of Population Health, NYU Grossman School of Medicine, New York, USA; 38 School of Exercise and Nutrition Sciences, Faculty of Health, Queensland University of Technology, Brisbane, Australia; 39 Institute for Medicine and Public Health, Vanderbilt University Medical Center, Nashville, USA; 40 School of Agriculture and Food Science, College of Health and Agricultural Sciences, University College Dublin, Dublin, Ireland; 41 Jacobs School of Medicine and Biomedical Sciences, University at Buffalo, Buffalo, USA; 42 Faculty of Health, Medicine and Life Sciences, Department of Health Promotion, Research Institute of Nutrition and Translational Research in Metabolism (NUTRIM) and Care and Public Health Research Institute (CAPHRI), Maastricht University, Maastricht, the Netherlands; 43 Department of Surgery, Dentistry, Pediatrics, and Gynecology, University of Verona, Verona, Italy; 44 Department of Preventive Medicine, University of Southern California, Los Angeles, USA; 45 Cedars-Sinai Medical Center, Los Angeles, USA; 46 Department of Health Sciences, Faculty of Medicine, Health and Human Sciences, Macquarie University, Macquarie Park, Australia; 47 Children’s Hospital of Philadelphia, Philadelphia, USA; 48 Graduate Program in Pediatrics, Child and Adolescent Health, Federal University of Health Sciences of Porto Alegre, Porto Alegre, Brazil

**Keywords:** Infant feeding, Diet, Movement, Sleep, Behaviour change techniques, Intervention components, Infants

## Abstract

**Background:**

Early childhood obesity prevention interventions that aim to change parent/caregiver practices related to infant (milk) feeding, food provision and parent feeding, movement (including activity, sedentary behaviour) and/or sleep health (i.e. target parental behaviour domains) are diverse and heterogeneously reported. We aimed to 1) systematically characterise the target behaviours, delivery features, and Behaviour Change Techniques (BCTs) used in interventions in the international Transforming Obesity Prevention for CHILDren (TOPCHILD) Collaboration, and 2) explore similarities and differences in BCTs used in interventions by target behaviour domains.

**Methods:**

Annual systematic searches were performed in MEDLINE, Embase, Cochrane (CENTRAL), CINAHL, PsycINFO, and two clinical trial registries, from inception to February 2023. Trialists from eligible randomised controlled trials of parent-focused, behavioural early obesity prevention interventions shared unpublished intervention materials. Standardised approaches were used to code target behaviours, delivery features and BCTs in both published and unpublished intervention materials. Validation meetings confirmed coding with trialists. Narrative syntheses were performed.

**Results:**

Thirty-two trials reporting 37 active intervention arms were included. Interventions targeted a range of behaviours. The most frequent combination was targeting all parental behaviour domains (infant [milk] feeding, food provision and parent feeding, movement, sleep health; n[intervention arms] = 15/37). Delivery features varied considerably. Most interventions were delivered by a health professional (*n* = 26/36), included facilitator training (*n* = 31/36), and were interactive (*n* = 28/36). Overall, 49 of 93 unique BCTs were coded to at least one target behaviour domain. The most frequently coded BCTs were: *Instruction on how to perform a behaviour* (n[intervention arms, separated by domain] = 102), *Behavioural practice and rehearsal* (*n* = 85), *Information about health consequences* (*n* = 85), *Social support (unspecified)* (*n* = 84), and *Credible source* (*n* = 77). Similar BCTs were often used for each target behaviour domain.

**Conclusions:**

Our study provides the most comprehensive description of the behaviour change content of complex interventions targeting early childhood obesity prevention available to date. Our analysis revealed that interventions targeted multiple behaviour domains, with significant variation in delivery features. Despite the diverse range of BCTs coded, five BCTs were consistently identified across domains, though certain BCTs were more prevalent in specific domains. These findings can be used to examine effectiveness of components and inform intervention development and evaluation in future trials.

**Trial registration:**

PROSPERO registration no. CRD42020177408.

**Supplementary Information:**

The online version contains supplementary material available at 10.1186/s12966-025-01708-9.

## Background

Health behaviours related to diet, movement (including physical activity, sedentary behaviour, screen time) and sleep are established early in life, and often continue throughout life to influence later childhood, adolescence and adult behavioural habits and associated health outcomes [1–5]. Such behaviours also influence obesity risk [6]. Given the early origins of health behaviours, interventions that commence in pregnancy or infancy provide an opportunity to establish healthy behavioural trajectories, preventing obesity and supporting healthy growth, with the potential to prevent adult-onset chronic conditions and extend health span [5, 7].

Infancy and early childhood are the periods when parents/caregivers (hereon referred to as parents) have the most influence on children’s health behaviours [8–10]. Parents can shape children’s behaviours through their knowledge, skills, values and opportunities and challenges within the home environment [11, 12]. Understanding the behaviour change process in the first 1000 days (i.e., conception to two years after birth) is a complex task. Parents’ behaviours need to adapt in response to children’s rapid development during this period. The behaviours parents enact result in changes to infants’ exposure (e.g., home activity environment, encouraging “tummy time”), to ultimately change infants’ behaviours (e.g., amount of active play) and later outcomes (e.g., obesity risk) [13].

Over the past 30 years, the important role of parents in influencing child health has resulted in many interventions designed to support parents in the first 1000 days [14]. The growing number of interventions within this population provide copious data that can be used to examine *how* parent-focused behavioural interventions may change parent behaviours [15] to determine whether they work, and for which populations they work [14]. This led to the formation of the Transforming Obesity Prevention in CHILDren (TOPCHILD) Collaboration [16]. The TOPCHILD Collaboration seeks to address these questions, by bringing together international researchers who are investigating parent-focused behavioural interventions commencing in pregnancy or the first 12 months after birth.

The nature of the target population (parents of young children) and varying types of behaviour change or maintenance required often results in highly complex interventions, targeting multiple behaviours over varying periods of time including over different developmental stages. A key challenge with complex, multicomponent interventions is describing what specific content these interventions actually include. The components of behaviour change interventions are generally underspecified in published reports, thus contributing to a poor understanding of how these interventions may influence behaviour [17], in turn limiting reproducibility, evidence synthesis and translation. Our present study focuses on examining *how* parent-focused behavioural interventions are delivered and *how* they aim to change or maintain behaviours for optimal diet, movement and sleep, regardless of their effects. Several checklists, taxonomies and ontologies have been developed that allow researchers to identify and separate components of complex interventions using a consistent language to describe, synthesise and compare interventions [18–20]. Systematic use of intervention coding can reveal important information about parental behaviours targeted for change, how an intervention was delivered (i.e. delivery features), and behaviour change techniques (BCTs; i.e. smallest, measurable and reproducible behaviour change components) used to change parents’ behaviours [18, 21]. Understanding this ‘black box’ of intervention components is a crucial step to allow replication and/or identify drivers of change.

Previous systematic reviews have begun to unpack this complexity primarily by examining the BCTs used in single behaviour domain (i.e., infant feeding alone) or a multi-component intervention overall (i.e., aggregated obesity prevention interventions regardless of behaviour) [22–27]. Thus, past reviews have limited information about interventions targeting different behaviour domain, including infant (milk) feeding, food provision, movement and sleep (alone or in combination). Without examination of intervention content by behaviour domain, we may not discover if different approaches are used or needed for certain types of behaviours. Such information is paramount for tailoring interventions to behaviours of greatest importance for different populations. Further, past reviews have relied on published intervention content descriptions that are often of limited depth. Our pilot study found 63% of BCTs were identified from unpublished intervention materials (e.g. facilitator manuals, participant resources) rather than published materials [23].

In this systematic review and intervention coding using published and unpublished materials from early childhood obesity prevention interventions, we sought to answer: 1) What are the target parental behaviours, delivery features and BCTs used in early childhood obesity prevention interventions?; and 2) What are the similarities and differences in BCTs used to target different parental behaviours?

## Methods

This study followed an intervention coding design using studies from the TOPCHILD Collaboration systematic review. Annual systematic searches were used to identify eligible trials, where investigators of eligible trials were invited to join the TOPCHILD Collaboration. This study is part of a series of complementary projects within the TOPCHILD Collaboration [16]. The protocol was prospectively registered (CRD42020177408) and published [15]. Reporting followed the Preferred Reporting Items for Systematic review and Meta-Analysis checklist [28] (Supplementary File 1), and guidance for reporting BCT Taxonomy was used [29]. Ethics approval was obtained from The University of Sydney Human Research Ethics Committee (project no. 2020/273) and Flinders University Social and Behavioural Research Ethics Committee (project no. HREC CIA2133-1).

### Eligibility criteria

Trials were eligible if they 1) were randomised controlled trials with a usual care control, no intervention or attentional control arm; 2) involved pregnant women or parents (including pregnant women) and their infant(s) aged 0 to 12 months at baseline; 3) evaluated child obesity prevention focused interventions that continued beyond pregnancy, and included at least one behavioural component related to infant (milk) feeding, food provision, movement (including physical activity, sedentary behaviour, screen time) or sleep; and 4) included at least one measure of child adiposity post-intervention. Trials were excluded if they focused solely on maternal obesity in pregnancy or included only non-behavioural interventions (e.g. supplements). While eligible interventions could commence antenatally, this study focused on understanding the behavioural content relating to parental behaviours directed towards infants, rather than focusing on parents’ own health behaviours.

### Information sources and search strategy

Systematic searches were conducted annually to identify eligible trials. The latest systematic search was performed on 27 February 2023 in the following databases from inception: Medline (Ovid), Embase (Ovid), Cochrane Central Register of Controlled Trials (CENTRAL), CINAHL (EBSCO), PsycINFO, and 28 March 2023 for ClinicalTrials.gov and the World Health Organization’s International Clinical Trials Registry Platform. No limits were placed on publication date, language or study status (planned, ongoing, completed). A search strategy for Medline is presented in Supplementary File 2. Reference lists of reviews, known to the authors, of randomised controlled trials in childhood obesity prevention were searched for additional eligible trials. Collaborators also notified the research team of potentially eligible trials.

### Selection process

Study selection included two stages: 1) systematic screening, 2) collation of unpublished intervention materials. In the first stage, title/abstracts and full text articles were independently screened in duplicate from a pool of reviewers (KEH, ALS, AB, MA, SL, JGW, BJJ, JA, AM) against the eligibility criteria, in Covidence (Veritas Health Innovation, Melbourne Australia), with disagreements resolved by consulting a third reviewer. In stage 2, eligible trials were invited by email to nominate one to two representative/s to join the TOPCHILD Collaboration and to share unpublished intervention materials (e.g., facilitator manuals, participant handouts, telephone scripts, videos, Short Message Service content, app content). This involved completing a form outlining all materials used in the intervention, as well as key publications and reporting any stakeholders involved in the intervention design. The review team collated key published materials (e.g., trial registration, protocols, main results publications). Trials were only included in the current study if they were able to share unpublished intervention materials (i.e. completed the requirements of the two-stage approach).

### Data extraction and risk of bias

Two reviewers (from a pool of reviewers: KEH, ALS, AB, MA, SL, JGW, BJJ, JA, SM) independently extracted general trial characteristics (e.g., authors, publication date, number of intervention arms, intervention/s name, geographical location, stage of enrolment), into Microsoft Excel® (Microsoft Corporation, version 2402). Additional trial characteristics, outcome measures, and risk of bias assessments will be reported in a complementary review examining intervention effectiveness, for which individual participant data are currently being collated [14].

### Coding of target behaviours, delivery features and behaviour change techniques

Outcomes for this review were intervention components coded by the study team, namely target behaviours, delivery features and BCTs. A standardised coding procedure was followed with a brief training session for all delivery feature coders (BJJ, SP, HIL, AM). Both BCT coders (BJJ, SP) completed the University College London online training for the BCT Taxonomy v1 (BCTTv1) [30], SP with a psychology background and BJJ having experience in coding BCTs in past projects (e.g., [23, 31, 32]). Target behaviours, delivery features and BCTs from published materials were independently coded in duplicate. Coder agreement was calculated using percent agreement for target behaviours and delivery features, and using kappa and prevalence-adjusted bias-adjusted kappa (PABAK) statistics for BCTs [33]. Any discrepancies in coding were resolved through discussion between coders, or by a third coder (for delivery features only as there were more than two coders available). We intended to code BCTs from unpublished materials in duplicate. However, given the volume of materials, high levels of coder agreement and data sharing agreements (e.g., confidentiality agreements), we used a modified protocol. Intervention arms were stratified by number of target behaviours and volume of materials, to randomly sample 25% of intervention arms to be coded in duplicate, with remaining intervention arms coded by a single coder and checked by a second coder. Unpublished materials not available in English were translated using the Google Translate document function [34]; videos could not be translated. Translation of materials was confirmed with trial representatives. We developed and tested a novel validation process, where trial representatives reviewed the retrospective coding of their intervention/s to ensure it aligned with the intervention intent. Further details of the validation process and its evaluation are reported elsewhere [35]. In brief, where possible, a virtual meeting was organised for one coder (BJJ) to discuss the coding with the trial representative(s) and to minimise reliance on trialists’ knowledge of BCTs and coding frameworks. Through the validation meeting any areas of uncertainty in coding were clarified (including any translations or untranslated video content), and the final coding was confirmed.

*Target behaviours* were coded to capture the parental behaviour(s) addressed in each intervention. A list of specific behaviours was generated by the study team and presented in the published protocol [15]. Target behaviours were clustered into one of the four behaviour domains: 1) infant (milk) feeding practices, 2) food provision and parent feeding practices, 3) movement practices and 4) sleep health practices.

*Delivery features* refer to the characteristics of how an intervention is delivered. A coding framework of delivery features was developed based on items in the Template for Intervention Description and Replication (TIDieR) reporting checklist [20]. Additionally ontologies from the Human Behaviour Change Project [19] were used to code the *intervention setting* (Intervention Setting Ontology), *mode of delivery* (Mode of Delivery Ontology) and *source delivering the intervention* (Intervention Source Ontology). We made minor refinements to the coding framework presented in the published protocol [15] (Supplementary File 3). The theories and rationales guiding the interventions, as described by trial representatives, were categorised into three types (1) Behaviour change theories, 2) Theories, models and frameworks for intervention content, and 3) Intervention development process), guided by previous classifications [36, 37]. Trial representative reported stakeholders involved in the design of the intervention (e.g. parents, health professionals, graphic designers, language interpreters, health-literacy experts) were categorised based on commonly reported terms.

*Behaviour Change Techniques* were coded using the BCT Taxonomy version 1(BCTTv1) [18]. Our target population was parents, and behaviours of interest were the four parental behaviour domains. A codebook was developed for this study (Supplementary File 4). This was an iterative process, drawing on previous intervention coding in obesity prevention and expert knowledge of the study team (BJJ, PMC, SP) [38]. Standard coding procedures were followed; for example, the whole intervention description was read before coding, and BCTs were coded as a ‘Yes’, ‘Maybe’ or ‘No’ based on the depth of evidence [30, 39, 40]. Each identified BCT was coded to the relevant target behaviour domain/s, or if unclear to an ‘unspecified behaviour domain’. During coding we identified BCTs relating to unintended target behaviours (e.g., BCTs relating to sleep, when not coded as a target behaviour domain for that trial); this was discussed and resolved through validation meetings with trial representatives. Coding and extracts to evidence each BCT were recorded in Microsoft Excel. Interventions where trialists had reported BCTs were recoded by the review team to minimise coder bias, differentiate BCTs by target behaviour domains and the BCTTv1. We intended to code control arms for the presence of BCTs relevant to the target population and behaviours, however given the paucity of information available about ‘usual care’ arms this was not possible.

### Synthesis of results

Coding accuracy was compared by type of materials: 1) published materials, 2) unpublished materials, and 3) validation meeting with trial representatives. We found differences in the depth of information included in material types (i.e. typically limited detail in descriptions in published materials) consistent with previous research [35, 41, 42] that resulted in differences in the codes identified. Thus, we refined the main analysis sample to include only interventions that included all three material types. Sensitivity analyses were conducted including intervention arms that provided published and unpublished materials (i.e. sensitivity analysis sample), using all coding prior to validation meetings. For this review, unique intervention arms were the primary unit of analysis, referred to from hereon as ‘interventions’; the term ‘trial’ is used when referring to characteristics relating to the trial (that could include one or more intervention arms). To address the first research question, a structured summary was prepared to describe the frequency of target behaviour domains, delivery features and BCTs coded. To address the second research question, narrative comparisons of BCTs were made to explore the similarities and differences in BCTs coded to target each parental behaviour domain. All analyses were repeated with the sensitivity analysis sample.

## Results

### Study selection and characteristics

**Fig. 1:**
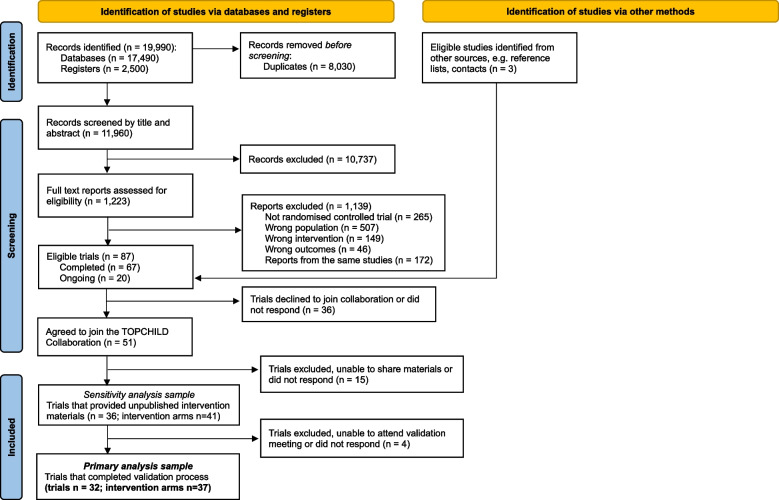
PRISMA flowchart showing search results of the TOPCHILD Collaboration

From the 11,960 records screened, 51 eligible trials joined the TOPCHILD Collaboration, of which 32 trials [43–74], comprising 37 intervention arms shared unpublished intervention materials and completed the validation process (Fig. 1). Trial characteristics are presented in Supplementary File 5. Trial start dates of included studies ranged from 2001 [48] to 2022 [75]. The majority of trials were completed at the time of coding (n[trials] = 28/32). Trials took place in nine countries, most frequently in the USA (*n* = 15/32), Australia (*n* = 6) and UK (*n* = 5), followed by New Zealand (*n* = 4), Norway (*n* = 3), Brazil (*n* = 2), Netherlands (*n* = 1), Spain (*n* = 1) and Sweden (*n* = 1). Trials mostly commenced in the first 6 months after birth (*n* = 16/32), or during pregnancy (*n* = 13), and most ended delivery of intervention content by child age of 12 months (*n* = 13/32) or 24 months (*n* = 9). Several interventions (n[interventions] = 15/37) also targeted parents’ own health behaviours (e.g. diet, movement, mental health), and three also targeted other child factors/behaviours (e.g., temperament/emotions).

### Parent behaviours targeted

**Fig. 2:**
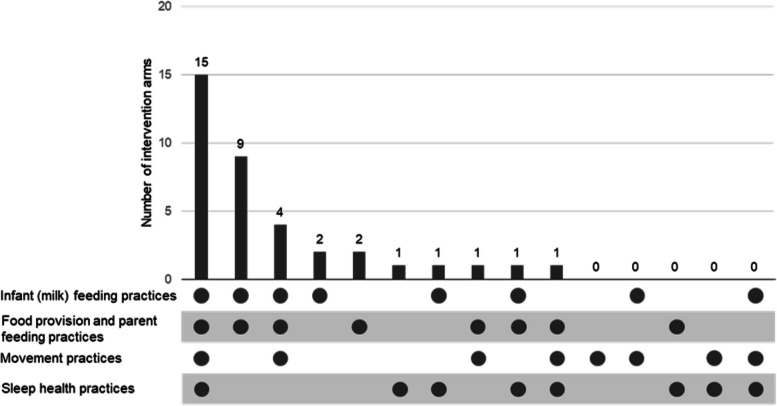
Frequency of combinations of target parental behaviour domains coded in early child obesity prevention interventions (*N* = 37)^a^. ^a^The x-axis details the possible combinations of the four target parental behaviour domains, with the dots indicating the domain is present in that combination. The y-axis indicates the number of interventions that targeted that combination of domains. Zeros represent that no intervention targeted the combination of domains

Interventions most commonly targeted food provision and parent feeding practices (*n* = 33/37); infant (milk) feeding practices (*n* = 32/37); followed by movement practices (*n* = 21/37), and sleep health practices (*n* = 19/37) (Table 1). Ten different combinations of target parental behaviour domains were identified from the possible 15 combinations (Fig. 2). The most common combination of domains identified was targeting all four domains (*n* = 15) [46, 47, 50, 53, 59, 60, 63, 64, 66, 67, 70–73], followed by a combination of infant (milk) feeding practices, and food provision and parent feeding practices (*n* = 9) [45, 48, 49, 52, 55, 62, 65, 68, 69], and a combination of infant (milk) feeding practices, food provision and parent feeding practices, and movement practices (*n* = 4) [43, 44, 53, 58].

**Table 1:** Frequency of specific target parental behaviours and domains coded in early child obesity prevention interventions^a^

**Target parental behaviour domain and specific target parental behaviours**	**Number of interventions (** ***N*** ** = 37)**
**Infant (milk) feeding practices**	**32**
Promoting and/or sustaining breastfeeding, including exclusive breastfeeding to 6 months of age	27
Feeding formula appropriately, if necessary (e.g. making formula per package instructions, feeding in response to the infant’s hunger/satiety cues, feeding with suitable types of formula)	25
Avoiding overfeeding, by not supplementing breastmilk with formula	16
Delaying introduction of solid foods (complementary feeding) until 6 months of age	28
**Food provision and parent feeding practices**	**33**
*Behaviours related to dietary intake*	
Providing appropriate types of foods (e.g. vegetables, meat and alternatives, fruits, whole grains, dairy)	33
Providing age-appropriate portions of each food group (i.e. portion sizes; incl. limiting portions of milk)	24
Limiting provision of certain foods and drinks (e.g. energy-dense, nutrient poor foods, sugar-sweetened beverages)	30
*Behaviours related to feeding practices*	
Offering foods repeatedly that have previously been rejected	29
Offering foods and drinks in response to infants’ hunger/satiety cues (e.g. letting the infant decide how much they eat, not pressuring to eat)	31
Avoiding use of food to control (or reward) the infant’s emotions, behaviour or consumption of other foods	26
Providing regular meal routines (incl. eating together, limiting distractions)	29
**Movement practices**	**21**
*Behaviours related to physical activity*	
Placing infant on their stomach for prone play (‘tummy time’)	20
Promoting age-appropriate physical activity such as active play, outdoor play, activities relating to fundamental movement skills	21
Providing toys that promote movement such as balls and toys on wheels	16
*Behaviours related to sedentary behaviour*	
Limiting the amount of time the infant is restrained (e.g. prams/strollers, high chairs, strapped on a caregivers back)	18
Limiting the amount of time the infant is exposed to screens (e.g. television, mobile devices)	21
Providing alternatives to screen time	20
**Sleep health practices**	**19**
Promoting regular sleep routine (e.g. calm, quiet, soothing)	19
Letting the infant settle back to sleep when stirring/crying during sleep cycle (e.g. leaving the room, only picking up infant when awake)	14
Promoting a positive sleep environment (e.g. quiet, darkened, warm)	16
Placing infant in cot/bassinet while awake and letting infant learn to fall asleep (e.g. following infant’s signs of tiredness)	16
Avoiding bed-sharing / co-sleeping (i.e. sleeping with the infant in the same bed)^b^	12
Maximising day-night differences (e.g. lights on and play in the day, lights off and sleep at night)	9

^a^*N* = 32 trials, reporting *n* = 37 unique intervention arms that completed the validation process. Interventions could target one or more behaviours. Average percent agreement between coders for target parental behaviour domains was 97% (range 75% to 100%)

^b^It was noted from validation meetings with trial representatives that bed-sharing is a cultural practice for some populations. Therefore, if bed-sharing was considered culturally appropriate, the intervention approach was to bed-shared safely

Each intervention targeted between two [61, 74] to 22 [47, 53, 66] specific parental behaviours, with an average of 13.5 (SD 6.5) behaviours per intervention. Table 1 presents the frequency of each specific parental behaviour.

### Delivery features

Given the heterogeneity and complexity of early childhood obesity prevention interventions, each delivery feature category was coded as present or absent, rather than categorising packages of intervention delivery (i.e. certain combinations of delivery features). One intervention did not complete validation of delivery features and was excluded from this analysis, resulting in 36 interventions available. Trial representatives reported if and what type of stakeholders were involved in the intervention design. In total, 30 of 32 trials reported any form of engagement with stakeholders in the intervention design. Stakeholders included parents (*n* = 25) [43, 44, 46–48, 50–57, 59, 61–64, 66–72, 74], health professionals (*n* = 22) [43, 44, 46, 47, 49–53, 55, 57–59, 61, 64, 67, 69–74], content experts (*n* = 8; e.g., paediatric researchers, experts in infant sleep) [46, 47, 58, 59, 64, 70, 72], graphic designers (*n* = 8) [46, 47, 50–53, 72, 73], health-literacy experts (*n* = 3) [50, 64, 72] and language interpreters (*n* = 2) [72, 74].

Table 2 presents the most commonly coded delivery features, see Supplementary File 6 for full details. Interventions ranged from having no underpinning theory in the intervention design process (*n* = 7) [48, 49, 62, 65] to being informed by multiple theories/frameworks/processes regardless of the theory type (2: *n* = 13, 3: *n* = 7, 4: *n* = 4; Supplementary File 6). There was large variation in the specific theories used for behaviour change, intervention content and intervention development. Six different theories of behaviour change were used, most frequently Social Cognitive Theory (*n* = 11) [50, 54, 57, 61, 63, 67, 70, 72, 73]/Social Learning Theory (*n* = 5) [46, 47, 57, 64], and the Health Belief Model (*n* = 4) [46, 47, 67]. For intervention content, 17 different theories, models and frameworks were reported, most commonly anticipatory guidance (*n* = 9) [43–45, 52–54, 73], responsive parenting (*n* = 5) [53, 56, 68, 70], and parenting support theory (*n* = 3) [43, 44, 73]. Three different intervention development processes were used, albeit rarely (three studies only), including Intervention Mapping (*n* = 2) [51, 58], the Behaviour Change Wheel (*n* = 1) [51], and the Model of Planned Promotion (*n* = 1) [56].

**Table 2:** Summary of the most common delivery features coded in early child obesity prevention interventions

**Delivery features**^a^	**Categories**	**Number of intervention (*n* = 36**^**b**^
**Why – theory: Rationale, theory or goal**	**Behaviour change theory**	
	Social Cognitive Theory	11
	Social Learning Theory	5
	Health Belief Model	4
	**Theories, models and frameworks for intervention content**	
	Anticipatory guidance	9
	Responsive parenting	5
	**Intervention development process**	
	Intervention Mapping	2
	**No theory used** (regardless of type)	7
**What – materials:** Physical or informational materials, including	Written materials	30
	DVD / video	16
	Tangible tools	10
	Other (e.g. social media group, magnets)	5
**What – procedures:** Procedures, activities, processed used in the intervention	Didactic sessions	30
	Peer/ facilitator support	27
	Interactive activities	16
	Group discussion	13
**Who provided – intervention delivered by**^**c**^**:** Expertise, background	**Health professional**	**26**
	Medical doctor	4
	Nursing and midwifery professional	16
	Other health professional (e.g. dietitians and nutritionist, physiotherapist)	13
	**Legal, social and cultural professional (e.g. psychologists)**	**4**
	**Health associate professional (e.g. community health workers)**	**3**
	**Higher education university student**	**3**
	**Not applicable**	**4**
**Training for the intervention:**	Yes	31
	No	1
	Not applicable	4
**How– delivery mode**^**d**^:	**Human interactional**	**29**
	Face to face	28
	**Printed material**	**29**
	Printed publication	27
	**Electronic**	**28**
	Call	17
	Playable electronic storage	9
	Mobile application	5
	Email	3
	Website	3
**Individual / Group:**	Individual (i.e., one-on-one)	31
	Group	15
**Unidirectional / Interactional**	Unidirectional	8
	Interactional	28
**Synchronous / Asynchronous**	Synchronous	31
	Asynchronous	10
**Where – intervention setting**^**e**^: Location	**Residential facility**	**23**
	Household residence^f^	23
	**Healthcare facility**	**11**
	Community healthcare facility	5
	**Educational facility**	5
	University facility	3
	**Community facility**	**5**
**When and how much – intervention dose**^**g**^:	Total intervention duration in weeks *(median, range)*	64.5 (0.3, 165.6)
	Total number of contacts *(median, range)*	9 (2, 105)
	Frequency of contact:	
	< Weekly	2
	Weekly to < monthly	7
	Monthly or greater	21
	Varied frequency	4
	Average duration of contact in hours (*median, range)*	7.5 (0.3, 30)
**Tailoring:** If the intervention was planned to be personalised, titrated or adapted at the participant level	Yes – included element of tailoring	27
	No	9
**Modifications:** If the intervention was modified during the study at the intervention level	Yes – intervention modified	8
	No	28
**Fidelity:** Planned and/or Actual	Yes	35
	No	1

Percent agreement between coders for delivery features was a mean 79% (SD 8%)

^a^Adapted from Hoffmann et al. [20] An intervention arm could be coded to multiple categories within a delivery feature domain

^b^One intervention arm in the primary analysis arm did not complete validation process for delivery features

^c^Intervention Source Ontology v3 [19] https://osf.io/zfn25/

^d^Mode of Delivery Ontology [19] https://osf.io/4j2xh/

^e^Intervention Setting Ontology v1 [19] https://osf.io/g8qfv/

^f^Household residence was assumed to be where participants accessed digital/remote intervention content (*n* = 5)

^g^Intervention duration reported in months were multiplied by 4.3 to estimate duration in weeks, (*n* = 1 missing data). Number, frequency and duration of contact for intervention delivery excluded data collection contact time (*n* = 2 missing data, where contact was variable and unable to be estimated)

Multiple types of materials and procedures were often used in a single intervention. Written materials (*n* = 30) [43–49, 52, 53, 55, 57–59, 61, 62, 62, 64–66, 68, 70–74] were the most frequently provided materials to participants, followed by DVD/videos (*n* = 16) [43, 44, 54, 56, 58, 59, 62–67, 70, 73, 74], and tangible tools (*n* = 10) [43–45, 58, 59, 62, 63, 72, 73] such as storybooks, balls, placemat and cups. Common procedures used to deliver intervention content were didactic sessions (*n* = 30; i.e., information provision) [45–49, 51–54, 56–58, 61–64, 66–73] and peer/facilitator support (*n* = 27) [43–50, 52, 53, 57–59, 61, 63–68, 71–74].

Intervention providers (i.e. facilitators) were relatively homogeneous across interventions. Interventions were predominately provided by health professionals (*n* = 26), including nursing and midwifery professionals (*n* = 16) [46, 47, 49, 50, 52, 53, 57, 59, 61, 62, 70], medical doctors (*n* = 4) [47, 49, 65, 72], and other health professionals (*n* = 13, such as dietitians and nutritionist, physiotherapist) [43–45, 50, 52, 53, 63, 66, 67, 71, 74]. Other types of facilitators included professionals related to health, such as psychologists (*n* = 3) [45, 50, 64], community health workers (*n* = 2) [58, 71] and higher education university students (*n* = 3, e.g. student dietitian) [48, 55, 68]. Four interventions were purely electronic without a facilitator [51, 54, 56, 69], and therefore not coded to a professional background nor was training applicable. Intervention providers received training in all but one study, using a facilitator.

The mode of delivery was highly varied, with interventions commonly using multiple modes of delivery (e.g. human interaction [in person], printed material and electronic, *n* = 19; human interaction and printed material, *n* = 8) (Supplementary File 6). The overall delivery modes were evenly split across human interaction (*n* = 29 [43–45, 47–50, 52, 53, 55, 57–59, 61, 62, 64–68, 70–74], predominately face-to-face *n* = 28), printed materials (*n* = 29) [43–49, 52, 53, 55, 57–59, 61, 62, 65–68, 70–74], and electronic (*n* = 28) [43, 44, 46, 47, 50–54, 56–59, 61–67, 69, 70, 73, 74]. Within electronic modes, website (*n* = 3) [54, 56, 69] and mobile applications (*n* = 5) [50, 51, 56, 63, 64] were less commonly coded. Most interventions were classified as interactional (*n* = 28) [43–50, 52, 53, 55–59, 61, 63, 64, 66–68, 70–74], were delivered synchronously (*n* = 31) [43–50, 52, 53, 55, 57–59, 61–68, 70–74] and included an individual (i.e. one-on-one) delivery approach (*n* = 31) [43, 44, 46–54, 56, 57, 61–63, 65–74].

Despite being predominantly delivered by health professionals, interventions were delivered in a range of settings, including healthcare facilities (*n* = 11) [43–45, 49, 57, 64–67, 72, 74], educational facilities (*n* = 5) [53, 55, 59, 68], community facilities (*n* = 5) [43, 43, 45, 57, 74] and research settings (*n* = 2) [61, 70]. Two thirds of interventions were delivered in the home (*n* = 23) [46–48, 50–53, 56, 57, 61–64, 69–71, 73]. Interventions were primarily delivered in one setting (*n* = 26, e.g. residential facility only *n* = 15), with ten interventions delivered in a combination of two settings (Supplementary File 6).

There was large variation in intervention dose as measured by duration of contact with the intervention content. The total number of contacts ranged from two [53, 55, 62] to 105 [63], across a total intervention duration of 2 days [55] to 39 months [57]. Contact frequency also varied, with monthly or greater frequency used in just over half of the interventions (*n* = 21) [43–49, 54, 57, 61, 62, 65, 67, 68, 70, 72, 73]. Total duration of contact per participant for intervention content ranged from an average of 18 min [69] to 30 h [58].

Three quarters of interventions (*n* = 27) [46–53, 57–59, 61–63, 66–68, 70–74] reported tailoring to the participant, often through individualised counselling. However, some interventions included screening and subsequent directing participants to additional resources/support [53]. Only eight interventions made modifications relating to the intervention content or delivery from what was initially planned [43, 44, 50, 59, 64–67]. Reasons for modifications often related to funding or COVID-19 pandemic restrictions. All but one intervention [65] reported planned or actual fidelity measures (*n* = 35); these varied but were commonly implementing standardised manuals or training, and in some interventions reviewing observations of intervention sessions or random fidelity audits (e.g. [57, 71, 72, 74]).

### Behaviour change techniques coded regardless of domain

Table 3 presents the frequently coded BCTs by target parental behaviour domain (see Supplementary File 7 for all BCTs), note one intervention could use the same BCT to target different parental behaviour domains (i.e. number of interventions per BCT can be greater than the total 37 interventions). Overall, 49 of the 93 unique BCTs were coded to at least one target parental behaviour domain, therefore, 44 possible BCTs were not identified in any intervention (Supplementary File 7, Table S4). The BCTTv1 is organised into 16 hierarchical clusters, and no identified BCT was coded in any behaviour domain to *Scheduled consequences* or *Covert learning* hierarchical BCT cluster. The most frequently (> 70% of interventions targeting the domain) coded BCTs regardless of target parental behaviour domain were: *4.1 Instruction on how to perform a behaviour* (*n* = 102), *8.1 Behavioural practice and rehearsal* (*n* = 85), *5.1 Information about health consequences* (*n* = 85), *3.1 Social support (unspecified)* (*n* = 84), and *9.1 Credible source* (*n* = 77).

**Table 3:** Frequency of commonly coded Behaviour Change Techniques in early child obesity prevention interventions (*N* = 37) by target parental behaviour domain^a^

BCT number and label	Infant (milk) feeding practices	Food provision and parent feeding practices	Movement practices	Sleep health practices	Overall (all behaviour domains tallied, *N* = 105)
	**(*n*** ** = 31)**	**(*n*** ** = 33)**	**(*n*** ** = 22)**	**(*n*** ** = 19)**	
**1. Goals and planning**					
1.1 Goal setting (behaviour)	15	14	13	9	51
1.2 Problem solving	25	22	13	9	69
1.4 Action planning	11	11	8	6	36
1.5 Review behaviour goal(s)	12	11	9	5	37
1.6 Discrepancy between current behaviour and goal	2	2	2	2	8
1.9 Commitment	2	2	2	0	6
**2. Feedback and monitoring**					
2.1 Monitoring of behaviour by others without feedback	0	1	0	0	1
2.2 Feedback on behaviour	9	6	6	4	25
2.3 Self-monitoring of behaviour	2	5	4	2	13
2.4 Self-monitoring of outcome(s) of behaviour	2	1	0	2	5
2.7 Feedback on outcome(s) of behaviour	7	5	4	2	18
**3. Social support**					
3.1 Social support (unspecified)	29	25	17	13	84
3.2 Social support (practical)	17	15	12	10	54
3.3 Social support (emotional)	7	5	2	1	15
**4. Shaping knowledge**					
4.1 Instruction on how to perform a behaviour	30	33	20	19	102
4.2 Information about antecedents	0	2	1	0	3
4.4 Behavioural experiments	1	3	2	3	9
**5. Natural consequences**					
5.1 Information about health consequences	27	29	20	9	85
5.2 Salience of consequences	0	5	0	0	5
5.3 Information about social and environmental consequences	11	11	10	1	33
5.6 Information about emotional consequences	0	3	3	2	8
**6. Comparison of behaviour**					
6.1 Demonstration of the behaviour	15	22	13	8	58
6.2 Social comparison	5	6	5	4	20
6.3 Information about others’ approval	0	1	0	0	1
**7. Associations**					
7.1 Prompts / cues	7	14	7	2	30
**8. Repetition and substitution**					
8.1 Behavioural practice / rehearsal	20	31	21	13	85
8.2 Behavioural substitution	10	16	13	4	43
8.3 Habit formation	1	13	6	9	29
8.4 Habit reversal	0	1	1	0	2
8.6 Generalisation of a target behaviour	0	2	2	2	6
8.7 Graded tasks	3	3	3	3	12
**9. Comparison of outcomes**					
9.1 Credible source	24	25	18	10	77
9.2 Pros and cons	8	2	2	1	13
9.3 Comparative imagining of future outcomes	0	1	0	0	1
**10. Reward and threat**					
10.3 Non-specific reward	2	2	2	2	8
10.4 Social reward	16	13	11	5	45
10.9 Self-reward	4	3	3	2	12
**11. Regulation**					
11.2 Reduce negative emotions	15	15	8	11	49
11.3 Conserving mental resources	0	2	0	0	2
**12. Antecedents**					
12.1 Restructuring the physical environment	1	14	14	5	34
12.2 Restructuring the social environment	3	12	11	4	30
12.3 Avoidance/ reducing exposure to cues for the behaviour	0	2	1	0	3
12.5 Adding objects to the environment	5	13	8	5	31
**13. Identity**					
13.1 Identification of self as role model	0	22	17	0	39
13.2 Framing / reframing	3	5	5	2	15
**15. Self-belief**					
15.1 Verbal persuasion about capability	10	9	6	4	29
15.2 Mental rehearsal of successful performance	0	0	1	0	1
15.3 Focus on past success	3	3	2	2	10
15.4 Self-talk	1	1	1	0	3

^a^Only BCTs that were coded to at least one intervention arm and target parental behaviour domain are displayed. No BCTs were coded from the *14. Scheduled consequences* or *16. Covert learning* hierarchical clusters. Note slightly different numbers from the target behaviours clusters (I *n* = 32, F *n* = 33, M *n* = 21, S *n* = 19, Table 1), as one intervention arm did not use any BCTs for that behaviour and one had unintendedly targeted a behaviour. Agreement between coders was high in both BCT coding of published materials (movement practices domain mean PABAK 0.88, SD 0.07 to sleep health practices domain mean 0.93, SD 0.05), and unpublished materials (movement practices domain mean 0.83, SD 0.08 to unspecified target behaviour domain mean 0.93, SD 0.04)

### Comparison of behaviour change techniques coded to target different parental behaviour domains

There were typically fewer BCTs per intervention coded to target sleep health practices (median 7, range 2 to 18), compared with the other target behaviour domains (infant [milk] feeding practices median 12, range 3 to 20; food provision and parent feeding practices median 12, range 3 to 32; movement practices median 13.5, range 2 to 29). Table 4 showcases examples of selected BCTs for relevant target behaviour domains.

**Table 4:** Examples of how selected BCTs were operationalised in early child obesity prevention interventions

**BCT**	**Short definition in child obesity prevention intervention context**	**Examples of application in a child obesity prevention intervention context**^**a**^
1.2 Problem solving	Parents identify factors impacting behaviour and select solutions	• The group format promoted discussion of strategies, successes and overcoming barriers to key messages • Extra home visit(s), phone or email contact involved providing specific individualized advice to address problems with breastfeeding (or formula feeding) • *‘Think about a tricky situation with your child. How do you want to respond? If you make a plan for this, it will work out better!’*
2.1 Monitoring of behaviour by others without feedback	Interventionists observe parent behaviours with the intent this will change behaviour but do not give feedback or advice	• At each visit, the nurse will spend time with the mother and infant, monitoring the parent–child feeding interaction and practice, and make note of their practices • *‘Did you buy more fruit, berries and vegetables last week? Do you think the family has eaten more than usual?’* [not in the context of data collection]
3.1 Social support (unspecified)	Provide general support or referral to further services/ resources	• Proactive telephone support will be provided between home visits to support behaviour maintenance and change • Families were encouraged to seek additional support with handouts providing with local support services • Intervention activities involved family members (i.e., infant father, grandparents, aunts and uncles)
4.1 Instruction on how to perform a behaviour	Information about recommendations and strategies	• Advice on breastfeeding establishment including length and number of feeds, positioning and attachment • The curriculum includes recommendations on establishing adequate sleep hygiene • *‘Let your child walk by themselves as much as possible. That way, they will exercise more and discover the world along the way.’*
5.1 Information about health consequences	Positive or negative health consequences of the behaviour	• Participants are shown a figure illustrating the relationship between healthy diet and health • *‘Continue to breastfeed your baby for as long as you both desire. Breastmilk continues to give your baby nutritional and health benefits – now and for life’* • *‘Tummy time and floor play with your baby will help with their physical and mental development’*
5.2 Salience of consequences	Use visuals to make the consequences of performing the behaviour more memorable	• Pictures of children’s teeth and gums with decay from putting children to bed with a bottle of milk • Videos of examples of pleasurable mealtimes and fun times playing with children
6.1 Demonstration of the behaviour	Image, video, or live demonstration of how to do the behaviour	• Instructional booklet with pictures demonstrating alternative ways of doing tummy time • Video of a parent modelling the responsive bottle feeding
6.3 Information about others’ approval	Provide parents with information on whether others will like, approve or disapprove of the behaviour	• Share details of other parents’ disapproval of giving infants mobile devices when in the pram • *‘Baby-led introduction to solids isn’t new – parents all over the world have used this approach for reasons such as: their baby wouldn’t let them feed them purées with a spoon, or their baby helped themselves to food off their plate.’*
8.1 Behavioural practice / rehearsal	Encourage repeating the behaviour	• The end of each session the facilitator summarises ‘things to practice at home’ • Parents attend a cooking class where they make healthy and nutritious meals for their infant • *‘Encourage practising active play with your child every day.’*
9.1 Credible source	Person with expertise or celebrity to persuade for or against the behaviour	• Home visits were provided by a specially trained community nurse • Sessions were co-led by a dietitian and exercise physiologist • Video includes a parent sharing their positive experience changing behaviour
9.3 Comparative imagining of future outcomes	Encourage parents to think about future outcomes of changing vs not changing a behaviour	• Participants were asked to imagine two situations of an unchanged and changed behaviour – imagine this situation at home…; you could…; discussion of favourable action • *‘Imagine this situation at home… You would like your toddler to try a new food. What would you do as they taste the new food?’*
11.2 Reduce negative emotions	Promote strategies to reduce negative emotions or stress	• *‘Try not to get stressed or upset if your child does not eat the new foods. The more you fuss about what you would like your child to eat, the more they will fuss and may have a negative experience with the food.’*
11.3 Conserving mental resources	Advise on how to reduce mental resources to performing a wanted behaviour	• The intervention provided a behaviour swap reference guide mobile device screen-saver with alternatives to screen time • *‘Write a list and keep on the fridge so that when your kids are asking for food you can easily be reminded of what healthy options you could offer.’*
13.1 Identification of self as role model	Promote parents as a role model	• Sessions highlighted the importance of parental role modelling health behaviours to children • *‘Be a good role model. Be active with your infant and limit your own screen use around them.’* • *‘Toddlers look to their caregivers to set positive examples for them such as eating and being active.’*
15.1 Verbal persuasion about capability	Telling parents they can change the behaviour	• *‘Learning about breastfeeding before birth can help boost your confidence. See ‘Breastfeeding your Baby’ booklet.’* • *‘You know your child and your family better than anyone – be confident in yourself as a parent and in your ability to influence your child’s sleep routine.’*
15.2 Mental rehearsal of successful performance	Advise parents to imagine themselves performing target behaviour successfully	• Activities include a mental task where parents are asked to imagine breastfeeding in different contexts • *‘Use visualisation strategies to imagine a scenario taking infant out to play or to a group.’*

^a^Examples are framed as they would be written in an intervention description, facilitator materials or directly to parents (as per the *italics*)

#### Infant (milk) feeding practices

Within this domain, there were 37 unique BCTs coded across all interventions. The most frequently coded BCTs were: *4.1 Instruction on how to perform a behaviour* (*n* = 30), *3.1 Social support (unspecified)* (*n* = 29), *5.1 Information about health consequences* (*n* = 27), *1.2 Problem solving* (*n* = 25), and *9.1 Credible source* (*n* = 24). There were no BCTs that were only coded to this domain.

#### Food provision and parent feeding practices

Within this domain, there were 48 unique BCTs coded across all interventions. The most frequently coded BCTs were: *4.1 Instruction on how to perform a behaviour* (*n* = 33), *8.1 Behavioural practice / rehearsal* (*n* = 31), *5.1 Information about health consequences* (*n* = 29), *3.1 Social support (unspecified)* (*n* = 25), and *9.1 Credible source* (*n* = 25). There were five BCTs that were only coded to this domain: *2.1 Monitoring of behaviour by others without feedback* (*n* = 1), *5.2 Salience of consequences* (*n* = 5), *6.3 Information about others’ approval* (*n* = 1), *9.3 Comparative imagining of future outcomes* (*n* = 1), and *11.3 Conserving mental resources* (*n* = 2).

#### Movement practices

Within this domain, there were 43 unique BCTs coded across all interventions. The most frequently coded BCTs were: *8.1 Behavioural practice / rehearsal* (*n* = 21), *4.1 Instruction on how to perform a behaviour* (*n* = 20), *5.1 Information about health consequences* (*n* = 20), *9.1 Credible source* (*n* = 18), *3.1 Social support (unspecified)* (*n* = 17), and *13.1 Identification of self as role model* (*n* = 17). There was one BCT only coded to this domain: *15.2 Mental rehearsal of successful performance* (*n* = 1).

#### Sleep health practices

Within this domain there were 37 unique BCTs coded across all interventions. All interventions included *4.1 Instruction on how to perform a behaviour* (*n* = 19). There were no other frequently coded BCTs (i.e. used in ≥ 70% of interventions), nor any BCTs that were only coded to this domain.

### Sensitivity analyses

Results from the sensitivity analyses performed on the dataset from coding published and unpublished materials, prior to refinements during the validation process with trial representatives (*N* = 41) are presented in Supplementary File 8. Key differences related to the presence of several intervention components being clarified in the validation meetings.

## Discussion

Interventions that aim to support parents’ practices to promote behaviours associated with healthy growth and obesity prevention in young children are varied and often complex. We sought to describe and compare the parent behaviours targeted, delivery features and BCTs of such interventions. We found it was common for interventions to target multiple behaviour domains and there was variation in most delivery features (e.g. theory, mode, provider, dose). While many different BCTs were coded, five BCTs were commonly identified regardless of target behaviour domain: *Instruction on how to perform a behaviour*, *Behavioural practice and rehearsal*, *Information about health consequences*, *Social support (unspecified)*, and *Credible source*. Although we found similar patterns in the coding of several types of BCTs across different target behaviour domains, some types of BCTs were more prevalent in certain behaviour domains.

### Components of early childhood obesity prevention interventions compared with other age groups

Most interventions in our review targeted multiple behaviour domains and related behaviours within each domain, with the most common combination targeting all four parental behaviour domains. Given the multiple influences on child growth [6] and that this is a period of rapid change in development, it is not surprising many interventions targeted multiple behaviours. While the multi-behaviour focus allows a comprehensive change approach to support healthy growth, it could also be perceived as overwhelming for participants. Reviews of obesity prevention interventions in older children (4–18 years), find similar results to the current review. Interventions in older children often target multiple behaviours, most often diet and movement related behaviours [76–79]. While few reviews report on sleep behaviours, one review of family-based interventions in children under 18 years reporting that sleep was only targeted in 20% of interventions [76]. There are broad similarities in the behavioural domains targeted across childhood, although few reviews in older children report on sleep behaviours.

We did, however, find variation in intervention complexity, ranging from interventions targeting multiple behaviours over many contacts, to brief interventions focused on one target behaviour domain. Combinations of delivery features used varied across the interventions. The features most often used were: written materials, information provision and peer/facilitator support; delivery by a health professional using multiple modes, interactional and individual components; single setting; duration of 15 months or longer with frequency of contacts monthly or more than monthly (e.g. quarterly); and elements of tailoring and fidelity measures. Existing reviews were limited in the breadth and depth of delivery features described, often only reporting theory use, intervention settings or duration [76–79], hence limit the comparisons that can be made with the current review findings. There were similarities in underpinning theories, with Social Cognitive/Learning Theory being the most used in studies in this review, in line with previous intervention findings in older age groups [76, 79]. Noting that the most common intervention settings differed between our review and those targeting 6–18-year-olds, which reflects our inclusion criteria of parent-focused interventions, but also the broader range of environments families interact with in later childhood and adolescence. The review by Hodder et al. 2022 [77], found interventions targeting 6–18-year-olds were less often delivered solely in the home only (6% vs our review 42%) or solely healthcare settings (2% vs 19%), more commonly delivered across multiple settings (49% vs 28%) or solely in school settings (32% vs 8%), compared with our sample. The setting differences are further supported by a review of family-based interventions [76], finding that interventions in young children were more often delivered at home (31%) and primary care settings (33%), compared to community and school settings (53% and 27%) used in interventions targeting older children. Unsurprisingly, we found emerging use of additional electronic modes, such as websites or mobile applications in several recent or ongoing interventions. Our finding aligns with a review by Ash et al. 2017 [76], who reported technology-based modes (i.e. computer, social media, text messages, internet) were more common in recent interventions. Taken together, these reviews reinforce the need for several delivery features to be tailored to the child age/parenting stage.

### Comparisons of BCTs by parent behaviour domain targeted

Comparisons of the types of BCTs coded to target each behavioural domain revealed similarities in the frequency of BCTs related to shaping knowledge, feedback, natural consequences, comparison of outcomes (e.g. credible source), regulation, and self-belief used in all/most domains. Many of these groups of BCTs relate to increasing parents’ capability through shaping knowledge and motivation through beliefs and persuasion [80]. Our findings likely reflect the commonly used self-regulation theories, for example needing to know how to do the behaviour, motivation for why change is needed, reducing stress impeding change, and/or building confidence in the ability to implement changes. Strategies relating to social and environmental opportunity were not as common in all domains, yet are important for behaviour change [80].

Notable differences were seen in BCTs relating to social support, with these being more common when targeting infant (milk) feeding practices. This reflects the type of behaviour, mostly breastfeeding, that may require additional support and resilience to implement [81]. Similarly, BCTs relating to goals/planning and rewards (i.e. social reward) were more commonly coded when targeting infant (milk) feeding practices or movement practices. There was variation in the specific BCTs identified, such as *Problem solving* being frequent in relation to breastfeeding, versus a range of goal focused BCTs coded in movement interventions. These findings are consistent with a review by Kassianos et al. [27] of interventions targeting breastfeeding, who reported BCTs relating to social support and *problem solving* were commonly coded across time intervals (birth-4wks, 5-8wks, 9-12wks, ≥ 13wks), with the BCT *Social support (unspecified*) associated with intervention effectiveness (at 5-8wks).

Several types of BCTs were more frequently identified to target both food provision and movement practices, than infant (milk) feeding practices or sleep health practices. Specifically, BCTs relating to comparison of behaviour (e.g. demonstrations), associations (e.g. prompts), repetition and substitution, antecedents (e.g. environment changes) and identity (e.g. role modelling). The grouping of techniques aligns with the repeated nature of these behaviours across the day and through developmental stages in early childhood [82, 83]. For example, with movement behaviours parents need to adjust the ‘how to’ strategies as an infant becomes more mobile and acquires new motor skills. Additionally, the strategies may reflect that diet and movement behaviours include both start/increase (e.g., increasing physical activity) and stop/decrease (e.g. decreasing sedentary behaviour) behaviours that require multiple behaviour change strategies [84, 85].

It was somewhat surprising many of the common types of BCTs coded in food provision and movement practices (e.g. comparison of behaviour, repetition, antecedents) were not as frequently coded when targeting sleep health behaviours, when similar challenges of adjusting strategies through developmental stages apply. However, there were substantially fewer BCTs targeting sleep health practices per intervention (median 7), than other behaviour domains (medians ranging from 12 to13.5), likely influencing this finding. The fact that these types of BCTs were identified in some interventions in our sample provide some support for the suitability/feasibility of use and evaluation of these techniques in a sleep context. The exception was BCT *13.1 Identification of self as a role model*, which is not practical to model for sleep health practices. Our findings suggest many similar techniques are used by intervention designers to target specific parent behaviours and across behaviour domains.

### Strengths and limitations

Our systematic search was a key strength, mitigating publication bias by searching clinical trial registries, and contacting and interviewing authors to clarify availability of outcomes and intervention materials. Annual search updates allowed for inclusion of the latest information about intervention approaches. We used multiple information sources including unpublished materials and validation with trial representatives, a considerable advantage over relying on the brief published descriptions typical of the field (e.g. [26, 27]). Using multiple information sources increased the alignment of intervention coding with the intent and content delivered [35]—a common limitation of retrospective coding. Standardised coding tools and high levels of coder agreement supported our assessment of intervention components, and our coding of BCTs to the target behaviour domains was comprehensive.

There are also some limitations to consider. Analysis did not include all identified eligible trials due to limited depth of information for coding. Nor did the current manuscript include reporting of trial results or risk of bias assessments as these were not related to the research questions and are reported in a complementary individual participant data meta-analysis [14]. Coding of BCTs focused on whether BCTs were present or absent and thus did not capture the dose or fidelity of components. Further, due to data availability and heterogeneity we were unable to explore parental engagement or acceptability of components. In addition, the BCTTv1 has recently been superseded by a BCT ontology that expands numerous BCTs to a total of 281 BCTs organised into 20 higher-level groups [86]. However, the BCTTv1 used in our review can be mapped to the new ontology for future intervention design [87]. The current review findings characterise what intervention designers have selected as potentially important components to change the parent behaviours targeted, often informed by engagement with parents in the intervention design and in some instances by behaviour change theory. Hence, there other untested components that may also be important to change the parent behaviours targeted.

### Implications for future research and practice

The current project provides crucial information on the components (i.e. target behaviours, delivery features and BCTs) and complexity of early childhood obesity prevention interventions. Our description of intervention components provides intervention designers with insights into existing and novel approaches used to inform design of future interventions to support parents in the first 1000 days. Our components evidence base can be used with intervention outcomes to conduct exploratory analyses on the effectiveness of common intervention components. We planned to do such analyses but were unable to, given the lack of harmonised aggregate data measuring obesity risk at the time [15]. Our complementary individual participant data meta-analysis seeking to determine *whether* interventions are effective and *for whom*, will overcome this issue by harmonising outcomes using raw datasets [14]. We will begin examining certain delivery features (i.e. intervention mode, setting, dose) and child behaviours and weight trajectories in the complementary review to explore *why* interventions may or may not change behaviour and growth [14]. Our future research plans to examine additional intervention components and child health behaviour and growth outcomes.

Our detailed analysis by target behaviour domain provides opportunities to explore a higher degree of tailoring of interventions. Tailoring could be based on health behaviour screening to determine behaviours of most importance for specific families [88], or through greater application of adaptive intervention methods [89]. Further, there is a need to assess the feasibility and acceptability of intervention components from a service, practitioner/facilitator, and parent perspective to inform translation of findings into policy and practice settings. One aspect of feasibility includes quantifying the costs to deliver such intervention components, which is a common consideration from a practice context [90]. In addition, there are opportunities to test untapped BCTs (including many rarely used and additional 22 not yet used), target behaviour combinations and delivery features in future interventions, ideally using factorial designs to determine optimal intervention packages [91] and monitoring the fidelity of BCTs delivered and received by participants [92, 93].

## Conclusions

Our systematic collation and detailed coding of published and unpublished intervention materials provides the most comprehensive description of the parental behaviours targeted, delivery features and BCTs identified in parent-focussed early childhood obesity prevention interventions to date. Our rich coding and description reveal the components within interventions, to provide direction for the design of future interventions to draw on commonly used components in existing interventions and gaps in underused intervention components (e.g. targeting sleep, certain BCTs). The findings also provide a synthesised evidence base to enable future exploration of components with intervention effects to inform the design of next generation interventions, policies and practice.

## Supplementary Information

Supplementary Material 1.

## Data Availability

The datasets used and/or analysed during the current study are available from the corresponding author following approval process from the TOPCHILD Collaboration.

## Abbreviations

BCT: Behaviour change technique
BCTTv1: Behaviour change technique taxonomy version 1
PABAK: Prevalence and bias adjusted kappa
