# Supplementary material for: Behavioural components and delivery features of early childhood obesity prevention interventions: intervention coding of studies in the TOPCHILD Collaboration systematic review
Source: Int J Behav Nutr Phys Act. 2025 Feb 5;22:14. doi: 10.1186/s12966-025-01708-9 (PMC11796048; doi:10.1186/s12966-025-01708-9)
Supplement: Supplementary file 1 — Supplementary Material 1. [file 12966_2025_1708_MOESM1_ESM.docx]

**Supplementary Files**

**Supplementary File 1: Completed PRISMA Reporting Checklist**

**Supplementary File 2: Search String**

**Supplementary File 3: Modifications to the prespecified methods**

**Supplementary File 4: BCT codebook for the TOPCHILD Collaboration**

**Supplementary File 5: Study characteristics**

**Supplementary File 6: Comprehensive summary of delivery features**

**Supplementary File 7: Comprehensive summary of BCTs**

**Supplementary File 8: Sensitivity analyses**

**SUPPLEMENTARY FILES**

**Supplementary File 1: Completed PRISMA Reporting Checklist**

| **Section and Topic** | **Item #** | **Checklist item** | **Location where item is reported** |
| --- | --- | --- | --- |
| **TITLE** | | |  |
| Title | 1 | Identify the report as a systematic review. | Title page 1 |
| **ABSTRACT** | | |  |
| Abstract | 2 | See the PRISMA 2020 for Abstracts checklist. | Abstract 4-5 |
| **INTRODUCTION** | | |  |
| Rationale | 3 | Describe the rationale for the review in the context of existing knowledge. | Introduction, pg 6-8 |
| Objectives | 4 | Provide an explicit statement of the objective(s) or question(s) the review addresses. | Introduction, pg 8 |
| **METHODS** | | |  |
| Eligibility criteria | 5 | Specify the inclusion and exclusion criteria for the review and how studies were grouped for the syntheses. | Eligibility criteria, pg 9 |
| Information sources | 6 | Specify all databases, registers, websites, organisations, reference lists and other sources searched or consulted to identify studies. Specify the date when each source was last searched or consulted. | Information sources pg 9-10 |
| Search strategy | 7 | Present the full search strategies for all databases, registers and websites, including any filters and limits used. | Information sources pg 9,-10  supplementary file 2 |
| Selection process | 8 | Specify the methods used to decide whether a study met the inclusion criteria of the review, including how many reviewers screened each record and each report retrieved, whether they worked independently, and if applicable, details of automation tools used in the process. | Selection process pg 10 |
| Data collection process | 9 | Specify the methods used to collect data from reports, including how many reviewers collected data from each report, whether they worked independently, any processes for obtaining or confirming data from study investigators, and if applicable, details of automation tools used in the process. | Selection process, data extraction pg 10 |
| Data items | 10a | List and define all outcomes for which data were sought. Specify whether all results that were compatible with each outcome domain in each study were sought (e.g. for all measures, time points, analyses), and if not, the methods used to decide which results to collect. | Coding of target behaviours, delivery features and behaviour change techniques, Pg 10-13 |
|  | 10b | List and define all other variables for which data were sought (e.g. participant and intervention characteristics, funding sources). Describe any assumptions made about any missing or unclear information. | Coding of target behaviours, delivery features and behaviour change techniques, Pg 10-13 |
| Study risk of bias assessment | 11 | Specify the methods used to assess risk of bias in the included studies, including details of the tool(s) used, how many reviewers assessed each study and whether they worked independently, and if applicable, details of automation tools used in the process. | Data extraction and risk of bias, pg 10 |
| Effect measures | 12 | Specify for each outcome the effect measure(s) (e.g. risk ratio, mean difference) used in the synthesis or presentation of results. | Synthesis of results, pg 13 |
| Synthesis methods | 13a | Describe the processes used to decide which studies were eligible for each synthesis (e.g. tabulating the study intervention characteristics and comparing against the planned groups for each synthesis (item #5)). | Synthesis of results, pg 13 |
|  | 13b | Describe any methods required to prepare the data for presentation or synthesis, such as handling of missing summary statistics, or data conversions. | Coding of target behaviours, delivery features and behaviour change techniques, Synthesis of results, pg 10-13 |
|  | 13c | Describe any methods used to tabulate or visually display results of individual studies and syntheses. | Synthesis of results, pg 13 |
|  | 13d | Describe any methods used to synthesize results and provide a rationale for the choice(s). If meta-analysis was performed, describe the model(s), method(s) to identify the presence and extent of statistical heterogeneity, and software package(s) used. | Coding of target behaviours, delivery features and behaviour change techniques, Synthesis of results, pg 10-13 |
|  | 13e | Describe any methods used to explore possible causes of heterogeneity among study results (e.g. subgroup analysis, meta-regression). | N/A |
|  | 13f | Describe any sensitivity analyses conducted to assess robustness of the synthesized results. | Synthesis of results pg 13 |
| Reporting bias assessment | 14 | Describe any methods used to assess risk of bias due to missing results in a synthesis (arising from reporting biases). | N/A |
| Certainty assessment | 15 | Describe any methods used to assess certainty (or confidence) in the body of evidence for an outcome. | N/A |
| **RESULTS** | | |  |
| Study selection | 16a | Describe the results of the search and selection process, from the number of records identified in the search to the number of studies included in the review, ideally using a flow diagram. | Study selection and characteristics, Figure 1, pg 14 |
|  | 16b | Cite studies that might appear to meet the inclusion criteria, but which were excluded, and explain why they were excluded. | Figure 1 |
| Study characteristics | 17 | Cite each included study and present its characteristics. | Study selection and characteristics pg 14, Supplementary file 3, Table S1 |
| Risk of bias in studies | 18 | Present assessments of risk of bias for each included study. | N/A |
| Results of individual studies | 19 | For all outcomes, present, for each study: (a) summary statistics for each group (where appropriate) and (b) an effect estimate and its precision (e.g. confidence/credible interval), ideally using structured tables or plots. | N/A |
| Results of syntheses | 20a | For each synthesis, briefly summarise the characteristics and risk of bias among contributing studies. | Results Pg 14-21 |
|  | 20b | Present results of all statistical syntheses conducted. If meta-analysis was done, present for each the summary estimate and its precision (e.g. confidence/credible interval) and measures of statistical heterogeneity. If comparing groups, describe the direction of the effect. | Results Pg 14-21 |
|  | 20c | Present results of all investigations of possible causes of heterogeneity among study results. | Results Pg 14-21 |
|  | 20d | Present results of all sensitivity analyses conducted to assess the robustness of the synthesized results. | Supplementary file 6 |
| Reporting biases | 21 | Present assessments of risk of bias due to missing results (arising from reporting biases) for each synthesis assessed. | N/A |
| Certainty of evidence | 22 | Present assessments of certainty (or confidence) in the body of evidence for each outcome assessed. | N/A |
| **DISCUSSION** | | |  |
| Discussion | 23a | Provide a general interpretation of the results in the context of other evidence. | Discussion, pg 21-27 |
|  | 23b | Discuss any limitations of the evidence included in the review. | Strengths and limitations, pg 24 |
|  | 23c | Discuss any limitations of the review processes used. | Strengths and limitations, pg 25-26 |
|  | 23d | Discuss implications of the results for practice, policy, and future research. | Implications for future research and practice pg 26-27 |
| **OTHER INFORMATION** | | |  |
| Registration and protocol | 24a | Provide registration information for the review, including register name and registration number, or state that the review was not registered. | Title page |
|  | 24b | Indicate where the review protocol can be accessed, or state that a protocol was not prepared. | Methods, pg 8 |
|  | 24c | Describe and explain any amendments to information provided at registration or in the protocol. | N/A |
| Support | 25 | Describe sources of financial or non-financial support for the review, and the role of the funders or sponsors in the review. | Funding, pg 29 |
| Competing interests | 26 | Declare any competing interests of review authors. | Competing interests, pg 29 |
| Availability of data, code and other materials | 27 | Report which of the following are publicly available and where they can be found: template data collection forms; data extracted from included studies; data used for all analyses; analytic code; any other materials used in the review. | Supplementary files |

*From:*  Page MJ, McKenzie JE, Bossuyt PM, Boutron I, Hoffmann TC, Mulrow CD, et al. The PRISMA 2020 statement: an updated guideline for reporting systematic reviews. BMJ 2021;372:n71. doi: 10.1136/bmj.n71

**Supplementary File 2: Search String**

Medline Search Strategy:

Ovid MEDLINE(R) ALL 1946 to search date

1. pediatric obesity/
2. Weight Gain/
3. obes*.ti,ab
4. (weight gain).ti,ab
5. (overweight or over weight).ti,ab
6. weight change*.ti,ab
7. ((bmi or body mass index) adj2 (gain or loss or change)).ti,ab
8. 1 or 2 or 3 or 4 or 5 or 6 or 7
9. social support/
10. ((behaviour or behavior) and change).ti,ab
11. ((behavio?r*) adj (therapy or modif* or strateg* or intervention* or advice or program* or class* or counsel* or educat* or instruct* or teach* or train* or guidance or lesson* or workshop* or module* or consultation* or session*)).ti,ab
12. ((lifestyle or life style) adj (chang* or modif* or strateg* or intervention* or advice or program* or class* or counsel* or educat* or instruct* or teach* or train* or guidance or lesson* or workshop* or module* or consultation* or session*)).ti,ab
13. social support.ti,ab
14. (peer adj2 support).ti,ab
15. counsel?ing.ti,ab
16. education* adj1 (intervention* or program* or class* or counsel* or teach* or workshop* or module* or consultation* or session*)).ti,ab
17. home visit*.ti,ab
18. 9 or 10 or 11 or 12 or 13 or 14 or 15 or 16 or 17
19. exp Breastfeeding/
20. Infant Nutritional Physiological Phenomena/
21. Child Nutrition Sciences/
22. Infant Food/
23. ((child or toddler or infant$) adj1 (food or feeding or nutrition$)).tw.
24. ((responsive or complementary) adj1 feeding).ti,ab
25. ((diet* or nutrition) adj (modif* or strateg* or intervention* or advice or program* or class* or counsel* or educat* or instruct* or teach* or train* or guidance or lesson* or workshop* or module* or consultation* or session*)).ti,ab
26. (healthy eating).ti,ab
27. (fruit or vegetable*).ti,ab
28. (high fat* or low fat* or fatty food*).ti,ab
29. 19 or 20 or 21 or 22 or 23 or 24 or 25 or 26 or 27 or 28
30. exp Exercise/
31. exercis*.ti,ab
32. (physical activity or physical inactivity).ti,ab
33. sedentary behavio?r.ti,ab
34. (screen time).ti,ab
35. 30 or 31 or 32 or 33 or 34
36. Sleep/
37. Exp Primary prevention
38. exp Health Promotion/
39. exp Health Education/
40. prevention.mp
41. prevent*.ti,ab
42. (health promotion or health education or health communication).ti,ab
43. exp Obesity/pc (Prevention and Control)
44. exp Overweight/pc (Prevention & Control)
45. (obesity adj2 prevent*).ti,ab
46. (overweight adj2 prevent*).ti,ab
47. 37 or 38 or 39 or 40 or 41 or 42 or 43 or 44 or 45 or 46
48. 8 and (18 or 29 or 35 or 36) and 47
49. exp child/ or exp infant/
50. ((child* or infant* or baby or toddler* or pediatr* or paediatr*) not adolescen*).ti,ab
51. (pregnan* or antenatal or parent or parent$1 or care giver or caregiver or guardian or family or families or mother$1 or father$1).ti,ab
52. 49 or 50 or 51
53. 48 and 52
54. (exp animals/ not humans.sh.) or (rat or rats or mouse or mice or rodent*).ti.
55. 53 not 54
56. controlled clinical trial.pt.
57. randomi#ed.ti,ab.
58. randomly.ab.
59. (clinical trials as topic or controlled clinical trials as topic).sh.
60. trial.ti.
61. exp randomized controlled trial/ or exp randomized controlled trials as topic/
62. 56 or 57 or 58 or 59 or 60 or 61
63. 55 and 62

**Supplementary File 3: Modifications to the prespecified methods**

Summary of modifications to the prespecified methods

| **Planned method^a^** | **Modified method and rationale** |
| --- | --- |
| All materials will be coded by two independent coders, when possible, exceptions may include when unpublished materials are only available in languages other than English | We intended to also code BCTs from unpublished materials in duplicate. However, given the volume of materials, high levels of coder agreement and data sharing agreements (e.g., confidentiality agreements), we used a modified protocol. Intervention arms were stratified by number of target behaviours and volume of materials, to randomly sample 25% of intervention arms to be coded in duplicate, with remaining intervention arms coded by a single coder and checked by a second coder.  Project resources available required a modified approach. |
| Control arms will also be coded for the presence of BCTs relevant to the target population and behaviours, and only BCTs unique to the intervention arm will be used in the results synthesis. | We intended to code control arms for the presence of BCTs relevant to the target population and behaviours, however given the paucity of information available about ‘usual care’ arms this was not possible.  Noting only one trial had a control arm that included the target behaviours. |
| The standardised procedure will be used whenever possible, however where unpublished materials are provided in languages other than English a modified procedure will be followed, such as using one coder fluent in the required language resulting in a subset of unpublished materials from a trial being coded once. If necessary, translation services will be sought to ensure the intervention components can be appropriately coded. | Unpublished materials not available in English were translated using the Google Translate document function; videos could not be translated. Translation of materials was confirmed with trial representatives.  Project resources available required a modified approach. |
| Table 2 presented the prespecified delivery feature coding framework and example categories. | When commencing coder training, we realised the need for additional categories and made the following refinements to the coding framework:   - What – materials: added an ‘other – please specify’ - What – procedures: added an ‘other – please specify’ - Who provided: added training for the intervention facilitator (Y/N/NR, briefly describe) - How – delivery mode: added individual and group delivery (part of the ontology) - When and how much – intervention dose: duration of contact in mins/hours   During the validation meetings we made the following refinements to the coding framework:   - What – procedures: added a ‘interactive activities’ and renamed ‘peer support’ to ‘peer / facilitator support’   During analysis we made the following refinement to the coding framework:   - Why: The theories and rationales guiding the interventions, as described by trial representatives, were categorised into three types (1) Behaviour change theories, 2) Theories, models and frameworks for intervention content, and 3) Intervention development process), guided by previous classifications.   Wide variation in the type of theories/rationale required modified approach. |
| To address the third research question, exploratory analyses will be undertaken to provide preliminary information about the effectiveness of commonly used intervention components in reducing BMI z-scores at 2 years of age (±6 months). For this purpose, a meta-regression analysis will be performed for each commonly used intervention component (ie, used in five or more interventions), to compare infant BMI z-score for trials including the intervention component compared with trials not including the intervention component. Our proposed approach will take into account small sample sizes and importantly the variability of the observed effect sizes, however, will not be able to determine independent effects of each component. | Our components evidence base can be used with intervention outcomes to conduct exploratory analyses on the effectiveness of common intervention components, to help evaluate the strength of evidence and identify gaps in knowledge by determining which components are associated with changes in child health behaviours and obesity risk. We planned to do such analyses but were unable to, given the lack of harmonised aggregate data measuring obesity risk at the time. Our complementary individual participant data meta-analysis seeking to determine *whether* interventions are effective and *for whom*, will overcome this issue by harmonising outcomes using raw datasets. We will begin examining certain delivery features (i.e. intervention mode, setting, dose) and child behaviours and weight trajectories in the complementary review to explore *why* interventions may or may not change behaviour and growth. Our future research plans to examine additional intervention components and child health behaviour and growth outcomes.  Aggregate data of BMI z-score at 2 years of age reported in publications was only available for n=12 intervention, which was insufficient sample size for the planned analyses. |

^a^ Planned methods replicated from Johnson et al. 2022 (BMJ Open, doi:10.1136/bmjopen-2020-048165)

**Supplementary File 4: BCT codebook for the TOPCHILD Collaboration**

**Table 1:** BCTTv1 93 hierarchically-clustered techniques with examples for the TOPCHILD Collaboration^a^

| **No.** | **Label** | **Short definition in child obesity prevention intervention context** | **Examples in a child obesity prevention intervention context** |
| --- | --- | --- | --- |
| **1. Goals and planning** | |  |  |
| 1.1 | Goal setting (behaviour) | Parents set a behavioural goal | What two activity goals would you like to set for this coming week?  Example of a goal: set a time for 2-3 snacks each day  **Note:** Only code if there is evidence that parents have set or selected the goal(s) for themselves. E.g. **“**A good goal is for you to limit your screentime to two hours or less”, would be coded as maybe as it is not clear that the parent set the goal. |
| 1.2 | Problem solving | Parents identify factors impacting behaviour and select solutions | The group format promoted discussion of strategies, successes and overcoming barriers to key messages.  Program includes a brainstorming exercise to solve common problems.  **Note:** Active engagement of parents in problem solving must be evident to code. E.g. do not code if solutions are recommended as part of the intervention as a passive process. |
| 1.3 | Goal setting (outcome) | Set a goal that targets a change due to a wanted behaviour OR any goal set where intention is unclear | Example of outcome focussed goal – Setting a growth trajectory goal, e.g. for child weight to stay within two percentile bands of their height.  Example of an unspecified goal – strategies include progressive goal setting, where there is no mention of the focus behaviour or outcome. |
| 1.4 | Action planning | Planning the when, where, and/or how of performing of a wanted behaviour | What would you like to do when feeding your toddler? When will you try it? How? – this example may include setting a goal Providing a weekly planner for active play. |
| 1.5 | Review behaviour goal(s) | Review behavioural goals with parents | Goals may be reviewed at each session, e.g. What was it that you wanted to try? How well did it work? Why?  Discuss any physical activity goals that were put in place since the last visit. |
| 1.6 | Discrepancy between current behaviour and goal | Show parents the difference between their current behaviour and their goal | Participants were shown their child’s average screen use compared with their target limit and the age-related recommendations. |
| 1.7 | Review outcome goal(s) | Review outcome goals with parents | As per 1.5 Review behaviour goal(s) but outcome focussed or unspecified. |
| 1.8 | Behavioural contract | Written agreement of parents behavioural goal with parent and facilitator | At the end of the session the sleep specialist recorded an agreed sleep health goal for the next month, that participants signed. |
| 1.9 | Commitment | Parents state their commitment to changing a behaviour | Parents were asked to state their commitment to limiting screen use by completing the following sentence: I will ____ to limit my child’s exposure to screens.  **Note:** Criteria for coding commitment vs goal setting might be required (i.e., committing to behaviour change vs setting a goal). |
| **2. Feedback and monitoring** | |  |  |
| 2.1 | Monitoring of behaviour by others without feedback | Interventionists observe parent behaviours with the intent this will change behaviour but do not give feedback or advice | At each visit, the nurse will spend approximately one hour with the mother and infant, monitoring the parent-child feeding interaction and practice, and behaviours promoting physical activity/inactivity in the child, and make note of their practices.  **Do not code** monitoring as part of data collection. |
| 2.2 | Feedback on behaviour | Give parents feedback on their performance of a behaviour | Participants receive computer-tailored feedback regarding infant physical activity level. |
| 2.3 | Self-monitoring of behaviour | Providing parents with ways to monitor their parenting behaviours (e.g. promoting tummy time) | Keep a record of your time spent in ‘screen time’ that is not work related for the next week, using this sheet. It can be interesting to take a look at your own behaviours.  All intervention participants receive a workbook to monitor strategies attempted at home.  **Note:** Evidence of strong intention or recommendation to self-monitor must be present to code, e.g. workbooks, sheets or resources provided to facilitate self-monitoring.  **Do not code** monitoring as part of data collection only. |
| 2.4 | Self-monitoring of outcome(s) of behaviour | Providing parents with ways to monitor outcomes from changing their behaviours (e.g. growth) | *Growth monitoring is likely to only be part of data collection or performed by intervention staff.*  Participants were given a diary to monitoring infant’s temperament, parent wellbeing and energy of the parent.  **Do not code** monitoring as part of data collection only (e.g. sleep diaries, accelerometers). |
| 2.5 | Monitoring outcome(s) of behaviour by others without feedback | Observing or recording the outcomes from parents’ behaviours | *Growth monitoring is likely to only be part of data collection or providing feedback.*  **Note:** Could include monitoring of non-growth outcomes as for 2.4, but by others not parents.  **Do not code** monitoring as part of data collection only (e.g. sleep diaries, pedometers). |
| 2.6 | Biofeedback | N/A | *Unlikely to be used in early child obesity prevention interventions. Given child age unlikely to collect physiological or biochemical measures.* |
| 2.7 | Feedback on outcome(s) of behaviour | Give parents feedback on the outcome of their performance of a behaviour (e.g. growth) | The infants’ growth was plotted on sex-specific reference curves for length-for-age and weight-for-age percentiles, which were then interpreted, and the infant’s growth was discussed with the mother.  **Do not code** monitoring as part of data collection only. |
| **3. Social support** | |  |  |
| 3.1 | Social support (unspecified) | Provide general support or referral to further services/ resources | Use of group discussion and peer support.  Information was presented to the group, and they were asked to problem solve together.  Proactive telephone support will be provided between home visits to support behaviour maintenance and change.  Families were encouraged to seek additional support with handouts provided with local support services, dietitans etc.  **Note:** Evidence does not need to state support if it is clear there is support arranged/provided. Attending a group session itself does not automatically imply social support.  Referral to services outside of the intervention can be captured under 3.1 Social support (unspecified). |
| 3.2 | Social support (practical) | Encourage tangible practice support from other caregivers/ support network | Accept other's help with washing, meals, house work, … anything!  Participants were provided an information pack for family members and other carers to help build support environments, such as how carers can support mothers in the desired behaviours.  How dad can help with breastfeeding: providing drinks, snacks and help with cooking.  **Unpublished note:** Some interventions create handouts for partners on how to do specific tasks with baby. Can be included as practical social support. |
| 3.3 | Social support (emotional) | Provide or encourage emotional support from other caregivers/ support network | Sessions were structured to ensure participants feel supported through sharing experiences of adopting parenting practices.  **Note:** Support to attend sessions or support for caring would not be coded, whereas support for adopting behaviour practices as part of the intervention would be. |
| **4. Shaping knowledge** | |  |  |
| 4.1 | Instruction on how to perform a behaviour | Information about recommendations and strategies | Discuss introduction of solid foods guidelines, recommendations; behavioural signs baby is ready for solid foods, what to give baby.  Advice on breastfeeding establishment including length and number of feeds, positioning and attachment.  **Note:** This can include booklets providing information on ‘how to’. |
| 4.2 | Information about antecedents | Parents are given information about situations to improve their performance of a wanted behaviour | Participants were provided information about the common causes of sleep problems in infants (e.g. sleep environment).  **Note:** Emphasis is on factors that predict behaviour not consequences of behaviour. |
| 4.3 | Re-attribution | Provide alternative explanations to parents about for the perceived causes of their behaviour | If a parent attributes infants poor sleep patterns to being a difficult baby, suggest that it may be a lack of a bedtime routine. |
| 4.4 | Behavioural experiments | Establishing ways for parents to test new behaviours to see what will happen | Ask parents to monitor babies sleep patterns to identify causes of poor sleep, and then to change routine to see if it makes a difference. |
| **5. Natural consequences** | |  |  |
| 5.1 | Information about health consequences | Positive or negative health consequences of the behaviour | Being active and mobile assists growth and bone development.  Powerpoint slide shown to participants shows figure illustrating the relationship between feeding and health.  **Note:** Health consequences may be positive or negative. |
| 5.2 | Salience of consequences | Use visuals to make the consequences of performing the behaviour more memorable | Pictures of children’s teeth and gums with decay from putting children to bed with a bottle of milk.  Videos of examples of pleasurable mealtimes and fun times playing with children. |
| 5.3 | Information about social and environmental consequences | Positive or negative external social, environmental or unspecified consequences of the behaviour | Breastfeeding saves the environment by not using lots of plastic.  Most people in society are positive or at least indifferent about breastfeeding.  Example of an unspecified information about consequences: Breastfeeding is safe and convenient.  **Note:** Social and environmental consequences are less focussed on the individual and more outward looking. |
| 5.4 | Monitoring of emotional consequences | Prompt parents to assess their feelings after attempting a behaviour. | Encourage participants to record how they feel after practising an infant sleep routine. |
| 5.5 | Anticipated regret | Make parents aware of the possibility of future regret about performing unwanted behaviours | Parents are asked how they would feel if they do not establish bedtime routines now.  **Note:** Needs to include the concept of regret inducement, e.g. asking how parents would feel. |
| 5.6 | Information about emotional consequences | Positive or negative emotional outcomes of the behaviour | Having a structured bedtime routine your child will feel more content.  By repeatedly providing vegetables without having the expectation that children will eat them, you will feel less stressed as a parent.  **Note:** This BCT relates to informing people about any emotional consequence for anyone not just the target population. |
| **6. Comparison of behaviour** | |  |  |
| 6.1 | Demonstration of the behaviour | Image, video, or live demonstration of how to do the behaviour | Instructional booklet with pictures demonstrating alternative ways of doing tummy time.  Video of a parent modelling the desired feeding behaviour.  **Note:** Do not code parent role modelling to infant as the infant is the recipient of the BCT not the parent. |
| 6.2 | Social comparison | Encourage parents to compare their own behaviour to other parents’ behaviour | Small group discussion where parents share their parenting styles, feeding practices and how their child lets them know they are full or hungry.  Provide information on sleep patterns and length of most babies at certain ages, and crying patterns, ask parents to compare this with their child. |
| 6.3 | Information about others’ approval | Provide parents with information on whether others will like, approve or disapprove of the behaviour | Share details of other parents’ disapproval of giving infants mobile devices when in the pram. |
| **7. Associations** | |  |  |
| 7.1 | Prompts / cues | A physical or social cue to prompt behaviour in the same location | Participants were provided with refrigerator magnets with the key messages from each module.  **Do not code** this BCT for observing infant’s hunger/satiety cues.  **Unpublished note:** May include information that can be printed out and put up around to house to act as cues/fridge magnets etc. |
| 7.2 | Cue signalling reward | N/A | *Unlikely to be used in early child obesity prevention interventions.* |
| 7.3 | Reduce prompts/cues | Gradually withdraw prompts to perform the behaviour | Push notification reminders to promote active play opportunities decreased over the intervention period. |
| 7.4 | Remove access to the reward | Advise or arrange for parents to be separated from situations that reward unwanted behaviours. | Remove or switch off tv/mobile devices to help parents focus on the infant during active play time. |
| 7.5 | Remove aversive stimulus | Advise or arrange to remove of an aversive stimulus to facilitate behaviour change | Parents were encouraged to avoid/minimise stress if it leads to unhelpful parenting strategies at mealtimes. |
| 7.6 | Satiation | N/A | *Unlikely to be used in early child obesity prevention interventions.* |
| 7.7 | Exposure | Provide parents with systematic confrontation with a feared stimulus to reduce the response to a later encounter | Parents were encouraged to allow the child to self-soothe (controlled crying) when they wake during the night and reassured that there are no negative consequences of this behaviour.  **Do not code** repeated exposure as a feeding strategy. |
| 7.8 | Associative learning | Present parents with a neutral stimulus and a stimulus that prompts the target behaviour until the neutral stimulus also elicits that behaviour | Advice on routine actions that baby can learn to associate with sleep. |
| **8. Repetition and substitution** | |  |  |
| 8.1 | Behavioural practice / rehearsal | Encourage repeating the behaviour | At the end of each session the facilitator summaries ‘things to practice at home’.  Encourage practising active play with your child every day.  Parents attend a 2 day cooking course where they make healthy and nutritious meals for their infant that can be made at home.  **Note:** Code ‘8.1 Behavioural practice/ rehearsal’, if ‘4.1 Instruction on how to perform the behaviour’ is repeatedly coded in the context.  **Unpublished Note:** Handouts/booklets may contain promotion of activities/behaviours for parents to do or try (new recipes, methods of soothing baby etc.). |
| 8.2 | Behavioural substitution | Encourage swapping out one behaviour for another | Offer fresh fruit rather than juice and offer cow’s milk rather than toddler milks.  Offer active play inside or outside rather than time in front of the TV. |
| 8.3 | Habit formation | Encourage repeating the behaviour in the same context | Parents are encouraged to create a bedtime routine for their infant that they follow every night. |
| 8.4 | Habit reversal | Encourage repeating an alternative behaviour in the same context | To replace feeding the infant during night awakenings, parents are taught 5 soothing techniques and are instructed to use them during the night instead. |
| 8.5 | Overcorrection | N/A | *Unlikely to be used in early child obesity prevention interventions.* |
| 8.6 | Generalisation of a target behaviour | Advise parents to perform a wanted behaviour from one situation in another | Encourage parents to use the same bedtime routine for infants’ night sleeps (e.g. dark, cool room), as for day sleeps. |
| 8.7 | Graded tasks | Breaking down a wanted behaviour into easy tasks that become harder | Provide staged suggestions for increasing activity over time.  Sleep technique "camping out" involves moving further away from the baby every night. |
| **9. Comparison of outcomes** | |  |  |
| 9.1 | Credible source | Person with expertise or celebrity to persuade for or against the behaviour | Sessions were co-led by a dietitian and psychologist with paediatric experience.  Home visits were provided by a specially trained community nurse.  **Note:** There is an assumption that specialised health professionals were selected to deliver the intervention as they are a known credible source for health information.  Only code if it is clear it is a credible source. |
| 9.2 | Pros and cons | Encourage exploration of the positives and negatives of behaviour change | What are the rewards and challenges of letting your baby walk?  After reading out various responses to a situation ask parents to brainstorm the pros and cons of the parenting behaviour. |
| 9.3 | Comparative imagining of future outcomes | Encourage parents to think about future outcomes of changing vs not changing a behaviour | Participants were asked to imagine two situations of an unchanged and changed behaviour – imagine this situation at home…; you could…; discussion of favourable action. |
| **10. Reward and threat** | |  |  |
| 10.1 | Material incentive (behaviour) | Tell parents they will receive a gift to reward progress on performing a wanted behaviour | Participants are informed they will receive a voucher for every month they continue exclusively breastfeeding, until around 6 months of age.  **Do not code** incentives provided as a recruitment or retention strategy, i.e. those not dependent on behaviour change progress. |
| 10.2 | Material reward (behaviour) | N/A | *Unlikely to be used in early child obesity prevention interventions.* |
| 10.3 | Non-specific reward | Provide a reward for progress on performing a wanted behaviour | Parents are given a star on their performance chart every time they introduce a new vegetable to their child. |
| 10.4 | Social reward | Congratulate parent for performing a wanted behaviour | When reviewing screen time goals, parents are congratulated for progress towards goal. (Note, for this example would also code BCT 1.5).  Parents were praised based on their effort at setting up a sleep routine in the final group session. |
| 10.5 | Social incentive | Inform parents that they will get a verbal or non-verbal reward to doing or attempting a wanted behaviour | Parents are informed they will receive a certificate at the end of the course based on progress towards limiting their infant’s sedentary time. |
| 10.6 | Non-specific incentive | Inform parents that a reward will be delivered if and there has been effort and/or progress in performing the behaviour | Parents are informed there will be a reward for completing the program if they have demonstrated effort towards changing their parenting practices.  **Do not code** if reward is an incentive for participation in the study and not for effort or progress during the intervention (i.e. participants were rewarded for their time is incentive to participate and not based on their progress in the intervention). |
| 10.7 | Self-incentive | Help parents plan future rewards they can give themselves for completing and/or making progress towards a behaviour | Encourage parents to plan a reward for themselves if they meet their infant sleep related goals. |
| 10.8 | Incentive (outcome) | N/A | *Unlikely to be used in early child obesity prevention interventions.* |
| 10.9 | Self-reward | Encourage parents to praise themselves for completing a wanted behaviour | At the end of the program, parents are encouraged to reflect on their progress towards infant feeding goals and praise themselves. |
| 10.10 | Reward (outcome) | N/A | *Unlikely to be used in early child obesity prevention interventions.* |
| 10.11 | Future punishment | N/A | *Unlikely to be used in early child obesity prevention interventions (in infants <12mths).* |
| **11. Regulation** | |  |  |
| 11.1 | Pharmacological support | N/A | *Unlikely to be used in early child obesity prevention interventions.* |
| 11.2 | Reduce negative emotions | Promote strategies to reduce negative emotions or stress | Sessions reinforce parents to relax, to not fuss, when children refuse to eat a meal offered.  Information was provided on how to look after oneself.  Warmth and independence are the parenting styles that will support the development of positive feeding practices for you and positive eating habits for your child. Try to be neutral around food and manage your emotions other ways. |
| 11.3 | Conserving mental resources | Advise on how to reduce mental resources to performing a wanted behaviour | Provide a behaviour swap reference guide mobile device screen-saver with alternatives to screen time.  Write a list and keep on the refrigerator so that when your kids are asking for food you can easily be reminded of what healthy options you could offer. |
| 11.4 | Paradoxical instructions | N/A | *Unlikely to be used in early child obesity prevention interventions.* |
| **12. Antecedents** | |  |  |
| 12.1 | Restructuring the physical environment | Promote changes to the home physical environment | Buy these foods occasionally (red foods). That way they are not always in the house tempting you and you don’t have to say no to your children all the time. |
| 12.2 | Restructuring the social environment | Promote changes to the home social environment (e.g., family rules) | Set up family rules around screen use, so that all caregivers and children have the same expectations.  Participants were encouraged to have a bedtime routine so that parents can focus on one child at a time. |
| 12.3 | Avoidance/ reducing exposure to cues for the behaviour | Encourage reducing exposure to prompts for unhealthy behaviours | Restructuring the pantry to keep unhealthy foods out of sight. |
| 12.4 | Distraction | Altering parent’s attention to avoid triggers for unwanted behaviour. | Parents are advised to engage in other activities (such as reading a book) while using controlled crying to distract themselves from feeling guilty. |
| 12.5 | Adding objects to the environment | Intervention adds objects to the home to facilitate the behaviour | Refrigerator magnets with the key messages were provided. (Note in this example would also code BCT 7.1)  Families were given a ball to encourage opportunities for active play.  **Note:** Objects can be added to prompt behaviour while also facilitating behaviour, provided this was the intention of the objects added.  **Do not code** providing healthy foods to infants; if the intervention includes strategies to change home food availability code BCT 12.1. |
| 12.6 | Body changes | N/A | *Unlikely to be used in early child obesity prevention interventions.* |
| **13. Identity** | |  |  |
| 13.1 | Identification of self as role model | Promote parents as a role model | Be a good role model. Eat a variety of foods and eat with your child.  Sessions highlighted the importance of parental role modelling health behaviours to children.  Toddlers look to their caregivers to set positive examples for them in regard to lots of behaviours – eating is one of them. |
| 13.2 | Framing / reframing | Encourage parents to change their thoughts and feelings around performing a wanted behaviour | Think of introducing food as 'fun experience' and activity and outside time as 'fun activity for the whole family', rather than chores. |
| 13.3 | Incompatible beliefs | Make parents aware of differences between current and past behaviours to create discomfort. | Highlight use of unfavourable parenting practices that are not in their child’s best interest long term and self-identification of being a good parent. |
| 13.4 | Valued self-identity | Affirm parent’s identity by asking them to rate their personal strengths | Activity where participants rate their parenting strengths before setting behavioural goals. (Note would also code BCT 1.1) |
| 13.5 | Identity associated with changed behaviour | N/A | *Unlikely to be used in early child obesity prevention interventions.* |
| **14. Scheduled consequences** | |  |  |
| 14.1 | Behaviour cost | N/A | *Unlikely to be used in early child obesity prevention interventions.* |
| 14.2 | Punishment | N/A | *Unlikely to be used in early child obesity prevention interventions.* |
| 14.3 | Remove reward | N/A | *Unlikely to be used in early child obesity prevention interventions.* |
| 14.4 | Reward approximation | Rewarding parents for making progress towards a behaviour and gradually moving rewards closer to the desired behaviour. | Arrange a self-reward for any reduction in screen time, gradually requiring infant use of screen devices to meet the planned weekly screen time. (Note would also code BCT 10.9) |
| 14.5 | Rewarding completion | Provide parents with a reward upon completion of a wanted behaviour. | Arrange a self-reward for first eating together, then make self-reward contingent on limiting distractions at mealtimes, then on having established regular meal routines. |
| 14.6 | Situation-specific reward | N/A | *Unlikely to be used in early child obesity prevention interventions.* |
| 14.7 | Reward incompatible behaviour | N/A | *Unlikely to be used in early child obesity prevention interventions.* |
| 14.8 | Reward alternative behaviour | N/A | *Unlikely to be used in early child obesity prevention interventions.* |
| 14.9 | Reduce reward frequency | N/A | *Unlikely to be used in early child obesity prevention interventions.* |
| 14.10 | Remove punishment | N/A | *Unlikely to be used in early child obesity prevention interventions.* |
| **15. Self-belief** | |  |  |
| 15.1 | Verbal persuasion about capability | Telling parents they can change the behaviour | You know your child and your family better than anyone – be confident in yourself as a parent and in your ability to influence your child’s sleep routine.  **Note:** Although ‘verbal’ is stated in the BCT label, text can also be code as this BCT as long as it is clear there is persuasion (i.e. it does not need to be delivered verbally). |
| 15.2 | Mental rehearsal of successful performance | Advise parents to imagine themselves performing target behaviour successfully | Activities include a mental task where parents are asked to imagine breastfeeding in different contexts. |
| 15.3 | Focus on past success | Advise parents to think about their previous success at performing the behaviour | In each session parents are asked to reflect and share things that worked in the past to limit infants screen use. |
| 15.4 | Self-talk | Prompt positive self-talk (aloud or silently) before and during the behaviour | Encourage parents to use positive self-talk when introducing new foods, e.g. its okay if they don’t like broccoli yet, I can keep offering it and they may like it next time. |
| **16. Covert learning** | |  |  |
| 16.1 | Imaginary punishment | N/A | *Unlikely to be used in early child obesity prevention interventions.* |
| 16.2 | Imaginary reward | Parents imagine preforming a wanted behaviour and a pleasant consequence for completing the behaviour | Imagine breastfeeding in public and someone praising you. |
| 16.3 | Vicarious consequences | Advise parents to observe the positive and negative consequences others receive for completing the wanted behaviour | Asks parents to observe the positive impact of other parents performing the behaviour such distracting kids when they ask for treats rather than giving in. |

^a^ Columns 1-2 replicated from Michie et al. 2011. Column 4 includes excerpts from Seidler et al. 2020.

**Supplementary File 5: Study characteristics**

**Table S1:** Characteristics of studies included in primary or sensitivity analysis samples

| **Trial name, registration number, and number of obesity prevention intervention arms** | **Trial representatives and contacts** | **Trial status and start date** | **Country** | **Intervention period^b^** | **Intervention targets parents own health behaviours** | **Intervention targets additional parental behaviours^c^** |
| --- | --- | --- | --- | --- | --- | --- |
| InFANT(1)  ISRCTN81847050  Arms: 1 | Karen Campbell, Kylie Hesketh | Completed  Start date: 2008 | Australia | Start: In the first 6 months  End: Finishes by 24 months | Diet, movement | N/A |
| InFANT Extend(2)  ACTRN12611000386932  Arms: 1 | Karen Campbell, Kylie Hesketh | Completed  Start date: 2011 | Australia | Start: In the first 6 months  End: Finishes by 36 months | Diet, movement | N/A |
| NOURISH(3)  ACTRN12608000056392  Arms: 1 | Lynne Daniels, Rebecca Byrne | Completed  Start date: 2008 | Australia | Start: In the first 6 months  End: Finishes by 24 months | N/A | N/A |
| CHAT(4)  ACTRN12616001470482  Arms: 2 | Li Ming Wen  Sarah Taki | Completed  Start date: 2017 | Australia | Start: During pregnancy  End: Finishes by 12 months | Diet, movement, mental health | N/A |
| Healthy Beginnings(5)  ACTRN12607000168459  Arms: 1 | Li Ming Wen  Sarah Taki | Completed  Start date: 2007 | Australia | Start: During pregnancy  End: Finishes by 24 months | Diet, movement, mental health | N/A |
| Prevention of overweight in children younger than 2 years old^a^(6)  Not available  Arms: 1 | Vera Verbestel | Completed  Start date: 2008 | Belgium | Start: In the first 12 to 24 months  End: Finishes by 24 to 36 months depending on start age | N/A | N/A |
| 10 steps for healthy feeding infants(7)  NCT00629629  Arms: 1 | Márcia Vitolo, Paola Baratto, Julia Valmorbida | Completed  Start date: 2001 | Brazil | Start: In the first 6 months  End: Finishes by 12 months | N/A | N/A |
| 10 steps for healthy feeding of children younger than 2y(8)  NCT00635453  Arms: 1 | Márcia Vitolo, Paola Baratto, Julia Valmorbida | Completed  Start date: 2008 | Brazil | Start: During pregnancy  End: Finishes by 24 months | N/A | N/A |
| Bump2Baby and Me(9)  ACTRN12620001240932  Arms: 1 | Sharleen O’Reilly | Ongoing  Start date: 2021 | Ireland, Spain, Australia, United Kingdom | Start: During pregnancy  End: Finishes by 12 months | Diet, movement, mental health, navigating health care system | N/A |
| PROBIT^a^(10)  NCT03131284  Arms: 1 | Claudio Maffeis | Completed  Start date: 2014 | Italy | Start: In the first 6 months  End: Finishes by 24 months | N/A | N/A |
| Samen Happie(11)  NTR6938  Arms: 1 | Levie Karssen, Junilla Larsen | Completed  Start date: 2018 | Netherlands | Start: In the first 12 months  End: Finishes by 48 months | Mental health; general parenting | N/A |
| BLISS(12)  ACTRN12612001133820  Arms: 1 | Rachael Taylor, Anne-Louise Heath | Completed  Start date: 2012 | New Zealand | Start: During pregnancy  End: Finishes by 12 months | N/A | N/A |
| POInz(13)  NCT00892983  Arms: 3 | Rachael Taylor, Barry Taylor, Anne-Louise Heath | Completed  Start date: 2009 | New Zealand | Start: During pregnancy  End: Finishes by 24 months | N/A | N/A |
| Early Food for Future Health(14)  ISRCTN13601567  Arms: 1 | Christine Helle | Completed  Start date: 2016 | Norway | Start: In the first 6 months  End: Finishes at 12 months | N/A | N/A |
| First food for infants(15)  ISRCTN45864056  Arms: 1 | Nina Cecilie Øverby | Completed  Start date: 2011 | Norway | Start: In the first 6 months  End: At 6 months | N/A | N/A |
| The Food4toddlers study(16)  ISRCTN92980420  Arms: 1 | Margrethe Røed | Completed  Start date: 2015 | Norway | Start: In the first 12 months  End: Finishes by 24 months | N/A | N/A |
| PRIMROSE(17)  ISRCTN16991919  Arms: 1 | Finn Rasmussen, Ata Ghaderi | Completed  Start date: 2008 | Sweden | Start: In the first 12 months  End: Finishes by 48 months | Diet, movement | N/A |
| HAPPY(18)  ISRCTN56735429  Arms: 1 | Maria Bryant | Completed  Start date:  2012 | United Kingdom | Start: During pregnancy  End: Finishes by 12 months | Diet, movement, mental health, (and preparation for birth) | N/A |
| HENRY III(19)  ISRCTN16529380  Arms: 1 | Maria Bryant, Kim Roberts, Claire Farrell | Ongoing  Start date: 2022 | United Kingdom | Start: In the first 6 months to 5 years  End: Finishes by 18 months to 6 years depending on start age | Diet, movement, emotional wellbeing | Feelings/emotions |
| HENRY(20)  NCT03333733  Arms: 1 | Maria Bryant, Kim Roberts, Claire Farrell | Completed  Start date: 2017 | United Kingdom | Start: In the first 6 months to 5 years  End: Finishes by 18 months to 6 years depending on start age | Diet, movement, emotional wellbeing | Feedings/emotions |
| Baby Milk Trial(21)  ISRCTN2 Arms: 10814693 | Rajalakshmi Lakshman, Ken Ong | Completed  Start date: 2011 | United Kingdom | Start: In the first 6 months  End: Finishes by 6 months | N/A | N/A |
| SLIMTIME(22)  NCT00359242  Arms: 3 | Ian Paul, Stephanie Anzman-Frasca | Completed  Start date: 2006 | United States | Start: In the first 6 months  End: Finishes by 6 months | N/A | N/A |
| Baby-Act(23)  NCT03517891  Arms: 1 | Maribel Campos | Ongoing  Start date:  2018 | United States | Start: During pregnancy  End: Finishes by 12 months | N/A | N/A |
| In-home Obesity Prevention to Reach Low-income Infants^a^(24)  Not available  Arms: 1 | Kayla de la Haye, Sarah-Jeanne Salvy | Completed  Start date: not reported | United States | Start: During pregnancy  End: Finishes by 2 years | Diet, movement | N/A |
| Grow2Gether(25)  NCT02037490  Arms: 1 | Alexander Fiks, Janani Ramachandran | Completed  Start date: 2014 | United States | Start: During pregnancy  End: Finishes by 12 months | Maternal self-care | N/A |
| Preventing the Early Introduction of Juice and Sugar-Sweetened Beverages in Infants’ Diets(26)  Not available  Arms: 1 | Natalia Golova | Completed  Start date:  2017 | United States | Start: In the first 6 months  End: Finishes by 6 or 12 months depending on start age | N/A | N/A |
| PEARLS(27)  NCT01771133  Arms: 1 | Kaumudi Joshipura, Maribel Campos Rivera | Completed  Start date: 2013 | United States | Start: During pregnancy  End: Finishes by 6 months | Diet, movement | N/A |
| Starting Early Obesity Prevention Program(28)  NCT01541761  Arms: 1 | Mary Jo Messito, Rachel Gross | Completed  Start date: 2012 | United States | Start: During pregnancy  End: Finishes by 36 months | General parenting techniques | N/A |
| Healthy Start to Feeding(29)  NCT03597061  Arms: 1 | Cathleen Stough | Completed  Start date: 2018 | United States | Start: In the first 6 months  End: Finishes by 12 months | N/A | N/A |
| Pilot Testing of Baby Feed: A Web Application for Health Professionals and Parents to Improve Infant Diets^a^(30)  NCT05062824  Arms: 1 | Cristina Palacios | Ongoing  Start date: 2021 | United States | Start: In the first 6 to 12 months  End: Finishes by 6 to 12 months depending on start age | N/A | N/A |
| SMS trial(31)  NCT02903186  Arms: 1 | Cristina Palacios, Jinan Banna | Completed  Start date: 2016 | United States | Start: In the first 6 months  End: Finishes by 6 months | N/A | N/A |
| INSIGHT(32)  NCT01167270; NCT03555331 (long term follow up)  Arms: 1 | Ian Paul, Jennifer Savage | Completed  Start date: 2012 | United States | Start: In the first 6 months  End: Finishes by 36 months | Diet | Child temperament |
| Preventing Childhood Obesity Through Early Guidance(33)  NCT01905072  Arms: 1 | Elizabeth Reifsnider, David McCormick | Completed  Start date: 2012 | United States | Start: During pregnancy  End: Finishes by 12 months | N/A | N/A |
| Greenlight(34)  NCT01040897  Arms: 1 | Russell Rothman, Eliana Perrin | Completed  Start date: 2010 | United States | Start: In the first 6 months  End: Finishes at 2 years | N/A | N/A |
| Delta Healthy Sprouts(35)  NCT01746394  Arms: 1 | Jessica Thomson | Completed  Start date: 2013 | United States | Start: During pregnancy  End: Finishes by 12 months | Diet, movement | N/A |
| Promoting Responsive Bottle-Feeding Within WIC(36)  Not available  Arms: 1 | Alison Ventura, Shannon Whaley | Ongoing  Start date: 2019 | United States | Start: In the first 6 months  End: Finishes by 6 months | N/A | N/A |

^a^ Trial included in sensitivity analysis only as had not complete validation process at the time of analysis.

^b^ Intervention content start and end categorised where possible into starting during pregnancy, within 6 months, or 12 months after birth, and concluding by 6, 12, 24 or 48 months of child age.

^c^ Additional child behaviours refers to parental behaviours other than those related to infant milk feeding practices, food provision and parent feeding practices, movement practices or sleep health practices.

**Supplementary File 6: Comprehensive summary of delivery features**

**Table S2:** Comprehensive summary of delivery features used in early child obesity prevention interventions

| **Delivery features**^a^ | **Categories** | **Number of intervention arms (n=36^b^)** |
| --- | --- | --- |
| **Why – theory: Rational, theory or goal** | **Behaviour change theory** |  |
|  | Social Cognitive Theory | 11 |
|  | Social Learning Theory | 5 |
|  | Health Belief Model | 4 |
|  | Trans Theoretical Model | 3 |
|  | I-Change Model | 1 |
|  | Self-Determination Theory | 1 |
|  | No behaviour change theory used | 19 |
|  | **Theories, models and frameworks for intervention content** |  |
|  | Anticipatory guidance | 9 |
|  | Attachment theory | 2 |
|  | Behavioral and educational, ecological framework | 2 |
|  | Family partnership model | 1 |
|  | Health literacy principles | 1 |
|  | Health self-empowerment theory | 2 |
|  | Implementation intentions | 1 |
|  | Motivational interviewing | 2 |
|  | Parenting support theory | 3 |
|  | Policy, Systems and Environment strategies | 1 |
|  | Reciprocal triad of causation | 1 |
|  | Responsive feeding | 1 |
|  | Responsive parenting | 5 |
|  | Social ecological model | 1 |
|  | Solution-focus support | 1 |
|  | Theoretical Domains Framework | 1 |
|  | Value enhanced nutrition assessment | 1 |
|  | No theories, models and frameworks for  intervention content used | 14 |
|  | **Intervention development process** |  |
|  | Behaviour Change Wheel | 1 |
|  | Intervention Mapping | 2 |
|  | Model of Planned Promotion | 1 |
|  | No intervention development process used | 32 |
|  |  |  |
| **Total number of different theories used** | 1 | 5 |
|  | 2 | 13 |
|  | 3 | 7 |
|  | 4 | 4 |
|  | No theory of any type used | 7 |
| **What – materials:** Physical or | Written materials | 30 |
| informational materials, including | DVD / video | 16 |
| provided to participants | Newsletters | 2 |
|  | Website | 2 |
|  | Mobile application | 4 |
|  | Tangible tools | 10 |
|  | Other (e.g. social media group, magnets) | 5 |
| **What – procedures:** Procedures, | Didactic sessions | 30 |
| activities, processed used in the | Peer/ facilitator support | 27 |
| intervention | Interactive activities | 16 |
|  | Group discussion | 13 |
| **Who provided – intervention delivered** | **Health professional** | **26** |
| **by^c^: Expertise, background** | Medical doctor | **4** |
|  | Generalist medical practitioner | **1** |
|  | Specialist medical practitioner | **4** |
|  | Nursing and midwifery professional | **16** |
|  | Nursing professional | **11** |
|  | Midwifery professional | **1** |
|  | Other health professional | **13** |
|  | Physiotherapist | **1** |
|  | Dietitian and nutritionist | **11** |
|  | Health professionals not elsewhere classified | **2** |
|  | **Teaching professional** | **1** |
|  | Other teaching professional | **1** |
|  | **Legal, social and cultural professional** | **4** |
|  | Social and religious professional | **4** |
|  | Psychologist | **3** |
|  | Social work and counselling professional | **1** |
|  | **Health associate professional** | **3** |
|  | Nursing and midwifery associate professional | **1** |
|  | Nursing associate professional | **1** |
|  | Other health associate professional | **2** |
|  | Community health worker | **2** |
|  | **Legal, social, cultural and related associate professional** | **2** |
|  | Sport and fitness worker | **2** |
|  | **Personal care worker** | **1** |
|  | Child care worker and teachers aide | **1** |
|  | Child care worker | **1** |
|  | **Researcher not otherwise specified** | **1** |
|  | **Higher education university student** | **3** |
|  | Not applicable | **4** |
|  |  |  |
| **Training for the intervention:** | Yes | **31** |
|  | No | **1** |
|  | Not applicable | **4** |
| **How**  **– delivery mode^d^**: | **Human interactional** | 29 |
|  | Face to face | 28 |
|  | At-a-distance | 1 |
|  | **Printed material** | 29 |
|  | Letter | 2 |
|  | Printed publication | 27 |
|  | Labelling | 2 |
|  | **Electronic** | 28 |
|  | Call | 17 |
|  | Audio call | 11 |
|  | Video call | 4 |
|  | Messaging | 6 |
|  | Playable electronic storage | 9 |
|  | Mobile application | 5 |
|  | Email | 3 |
|  | Website | 3 |
| **Delivery mode combinations** | Electronic only | 5 |
|  | Human interaction + Printed material | 8 |
|  | Human interaction + Electronic | 2 |
|  | Printed material + Electronic | 2 |
|  | Human interaction + Printed material + Electronic | 19 |
|  |  |  |
| **Individual / Group:** | Individual (i.e., one-on-one) | 31 |
|  | Group | 15 |
|  |  |  |
| **Unidirectional / Interactional** | Unidirectional | 8 |
|  | Interactional | 28 |
|  |  |  |
| **Synchronous / Asynchronous** | Synchronous | 31 |
|  | Asynchronous | 10 |
| **Where – intervention setting^e^**: Location | **Residential facility** | 23 |
|  | Household residence^f^ | 23 |
|  | **Healthcare facility** | 11 |
|  | Hospital facility | 2 |
|  | Hospital outpatient clinic facility | 1 |
|  | Doctor-led primary care facility | 1 |
|  | Community healthcare facility | 5 |
|  | Community outpatient clinic facility | 1 |
|  | **Educational facility** | 5 |
|  | Early year facility | 1 |
|  | University facility | 3 |
|  | **Community facility** | 5 |
|  | Library facility | 2 |
|  | **Research facility** | 2 |
| **Intervention setting combinations** | Residential facility only | 15 |
|  | Healthcare facility only | 7 |
|  | Education facility only | 3 |
|  | Community facility only | 1 |
|  | Residential + Healthcare facilities | 2 |
|  | Residential + Educational facilities | 2 |
|  | Residential + Community facilities | 2 |
|  | Residential + Research facilities | 2 |
|  | Healthcare + Community facilities | 2 |
| **When and how much – intervention** | Total intervention duration in weeks *(median, range)* | 64.5 (0.3, 165.6) |
| **dose^g^**: | Total number of contacts *(median, range)* | 9 (2, 105) |
|  | Frequency of contact: |  |
|  | < Weekly | 2 |
|  | Weekly to < monthly | 7 |
|  | Monthly or greater | 21 |
|  | Varied frequency | 4 |
|  | Average duration of contact in hours (*median, range)* | 7.5 (0.3, 30) |
| **Tailoring: If the intervention was planned to be personalised, titrated or** | Yes – included element of tailoring | 27 |
| **adapted at the participant level** | No | 9 |
| **Modifications:** If the intervention was | Yes –intervention modified | 8 |
| modified during the study at the intervention level | No | 28 |
| **Fidelity:** Planned and/or Actual | Yes | 35 |
|  | No | 1 |

^a^ Adapted from Hoffmann et al.^36^ An intervention arm could be coded to multiple categories within a delivery feature cluster. One intervention arm in the primary analysis arm did not complete validation process for delivery features.

^b^ One intervention arm in the primary analysis arm did not complete validation process for delivery features.

^c^ Intervention Source Ontology v3(18) <https://osf.io/zfn25/>

^d^ Mode of Delivery Ontology(18) <https://osf.io/4j2xh/>

^e^ Intervention Setting Ontology v1(18) <https://osf.io/g8qfv/>

^f^ Household residence was assumed to be where participants accessed digital/remote intervention content (n=5).

^g^ Intervention duration reported in months were multiplied by 4.3 to estimate duration in weeks, (n=1 missing data). Number, frequency and duration of contact for intervention delivery excluded data collection contact time (n=2 missing data, where contact was variable and unable to be estimated).

**Supplementary File 7: Comprehensive summary of BCTs**

**Table S3:** Frequency of all coded Behaviour Change Techniques in early child obesity prevention interventions (N=37) by target parental behaviour domain^a^

| **BCT number and label** | **Infant (milk) feeding practices** | **Food provision and parent feeding practices** | **Movement practices** | **Sleep health practices** | **Overall**  **(all behaviour domains tallied, N=105)** |
| --- | --- | --- | --- | --- | --- |
|  | **(n=31)** | **(n=33)** | **(n=22)** | **(n=19)** |  |
| **1. Goals and planning** |  |  |  |  |  |
| 1.1 Goal setting (behaviour) | 15 | 14 | 13 | 9 | 51 |
| 1.2 Problem solving | 25 | 22 | 13 | 9 | 69 |
| 1.4 Action planning | 11 | 11 | 8 | 6 | 36 |
| 1.5 Review behaviour goal(s) | 12 | 11 | 9 | 5 | 37 |
| 1.6 Discrepancy between current behaviour and goal | 2 | 2 | 2 | 2 | 8 |
| 1.9 Commitment | 2 | 2 | 2 | 0 | 6 |
| **2. Feedback and monitoring** |  |  |  |  |  |
| 2.1 Monitoring of behaviour by others without feedback | 0 | 1 | 0 | 0 | 1 |
| 2.2 Feedback on behaviour | 9 | 6 | 6 | 4 | 25 |
| 2.3 Self-monitoring of behaviour | 2 | 5 | 4 | 2 | 13 |
| 2.4 Self-monitoring of outcome(s) of behaviour | 2 | 1 | 0 | 2 | 5 |
| 2.7 Feedback on outcome(s) of behaviour | 7 | 5 | 4 | 2 | 18 |
| **3. Social support** |  |  |  |  |  |
| 3.1 Social support (unspecified) | 29 | 25 | 17 | 13 | 84 |
| 3.2 Social support (practical) | 17 | 15 | 12 | 10 | 54 |
| 3.3 Social support (emotional) | 7 | 5 | 2 | 1 | 15 |
| **4. Shaping knowledge** |  |  |  |  |  |
| 4.1 Instruction on how to perform a behaviour | 30 | 33 | 20 | 19 | 102 |
| 4.2 Information about antecedents | 0 | 2 | 1 | 0 | 3 |
| 4.4 Behavioural experiments | 1 | 3 | 2 | 3 | 9 |
| **5. Natural consequences** |  |  |  |  |  |
| 5.1 Information about health consequences | 27 | 29 | 20 | 9 | 85 |
| 5.2 Salience of consequences | 0 | 5 | 0 | 0 | 5 |
| 5.3 Information about social and environmental consequences | 11 | 11 | 10 | 1 | 33 |
| 5.6 Information about emotional consequences | 0 | 3 | 3 | 2 | 8 |
| **6. Comparison of behaviour** |  |  |  |  |  |
| 6.1 Demonstration of the behaviour | 15 | 22 | 13 | 8 | 58 |
| 6.2 Social comparison | 5 | 6 | 5 | 4 | 20 |
| 6.3 Information about others’ approval | 0 | 1 | 0 | 0 | 1 |
| **7. Associations** |  |  |  |  |  |
| 7.1 Prompts / cues | 7 | 14 | 7 | 2 | 30 |
| **8. Repetition and substitution** |  |  |  |  |  |
| 8.1 Behavioural practice / rehearsal | 20 | 31 | 21 | 13 | 85 |
| 8.2 Behavioural substitution | 10 | 16 | 13 | 4 | 43 |
| 8.3 Habit formation | 1 | 13 | 6 | 9 | 29 |
| 8.4 Habit reversal | 0 | 1 | 1 | 0 | 2 |
| 8.6 Generalisation of a target behaviour | 0 | 2 | 2 | 2 | 6 |
| 8.7 Graded tasks | 3 | 3 | 3 | 3 | 12 |
| **9. Comparison of outcomes** |  |  |  |  |  |
| 9.1 Credible source | 24 | 25 | 18 | 10 | 77 |
| 9.2 Pros and cons | 8 | 2 | 2 | 1 | 13 |
| 9.3 Comparative imagining of future outcomes | 0 | 1 | 0 | 0 | 1 |
| **10. Reward and threat** |  |  |  |  |  |
| 10.3 Non-specific reward | 2 | 2 | 2 | 2 | 8 |
| 10.4 Social reward | 16 | 13 | 11 | 5 | 45 |
| 10.9 Self-reward | 4 | 3 | 3 | 2 | 12 |
| **11. Regulation** |  |  |  |  |  |
| 11.2 Reduce negative emotions | 15 | 15 | 8 | 11 | 49 |
| 11.3 Conserving mental resources | 0 | 2 | 0 | 0 | 2 |
| **12. Antecedents** |  |  |  |  |  |
| 12.1 Restructuring the physical environment | 1 | 14 | 14 | 5 | 34 |
| 12.2 Restructuring the social environment | 3 | 12 | 11 | 4 | 30 |
| 12.3 Avoidance/ reducing exposure to cues for the behaviour | 0 | 2 | 1 | 0 | 3 |
| 12.5 Adding objects to the environment | 5 | 13 | 8 | 5 | 31 |
| **13. Identity** |  |  |  |  |  |
| 13.1 Identification of self as role model | 0 | 22 | 17 | 0 | 39 |
| 13.2 Framing / reframing | 3 | 5 | 5 | 2 | 15 |
| **15. Self-belief** |  |  |  |  |  |
| 15.1 Verbal persuasion about capability | 10 | 9 | 6 | 4 | 29 |
| 15.2 Mental rehearsal of successful performance | 0 | 0 | 1 | 0 | 1 |
| 15.3 Focus on past success | 3 | 3 | 2 | 2 | 10 |
| 15.4 Self-talk | 1 | 1 | 1 | 0 | 3 |

^a^ Only BCTs that were coded to at least one intervention arm and target parental behaviour cluster are displayed. No BCTs were coded from the *14. Scheduled consequences* or *16. Covert learning* hierarchical clusters. Note slightly different numbers from the target behaviours clusters (IF n=32, FP n=33, M n=21, S n=19, Table 1), as one intervention arm did not use any BCTs for that behaviour and one had unintendedly targeted a behaviour.

**Table S4:** BCTs not coded to any domain or intervention, by likely relevance for early childhood obesity prevention interventions

| **BCTs not coded but possibly relevant** | **BCTs not coded but unlikely relevant** |
| --- | --- |
| 1.7 Review outcome goal(s) | 2.6 Biofeedback |
| 1.8 Behavioural contract | 7.2 Cue signalling reward |
| 2.5 Monitoring outcome(s) of behaviour by others without feedback | 7.6 Satiation |
| 4.3 Re-attribution | 8.5 Overcorrection |
| 5.4 Monitoring of emotional consequences | 10.2 Material reward (behaviour) |
| 5.5 Anticipated regret | 10.8 Incentive (outcome) |
| 7.3 Reduce prompts/cues | 10.10 Reward (outcome) |
| 7.4 Remove access to the reward | 10.11 Future punishment |
| 7.5 Remove aversive stimulus | 11.1 Pharmacological support |
| 7.7 Exposure | 11.4 Paradoxical instructions |
| 7.8 Associative learning | 12.6 Body changes |
| 10.1 Material incentive (behaviour) | 13.5 Identity associated with changed behaviour |
| 10.5 Social incentive | 14.1 Behaviour cost |
| 10.6 Non-specific incentive | 14.2 Punishment |
| 10.7 Self-incentive | 14.3 Remove reward |
| 12.4 Distraction | 14.6 Situation-specific reward |
| 13.3 Incompatible beliefs | 14.6 Situation-specific reward |
| 13.4 Valued self-identity | 14.7 Reward incompatible behaviour |
| 14.4 Reward approximation | 14.8 Reward alternative behaviour |
| 14.5 Rewarding completion | 14.9 Reduce reward frequency |
| 16.2 Imaginary reward | 14.10 Remove punishment |
| 16.3 Vicarious consequences | 16.1 Imaginary punishment |

**Table S5:** Number of total Behaviour Change Techniques per intervention arm, and target parental behaviour domain

|  | **Infant (milk) feeding practices** | **Food provision and parent feeding practices** | **Movement practices** | **Sleep health practices** |
| --- | --- | --- | --- | --- |
|  | **Median (Range)** | **Median (Range)** | **Median (Range)** | **Median (Range)** |
| Number of total BCTs per intervention arm | 12 (3, 20) | 12 (3, 32) | 13.5 (2, 29) | 7 (2, 18) |

**Supplementary File 8: Sensitivity analyses**

Sensitivity analyses were performed on the dataset from coding published and unpublished materials, prior to refinements during the validation process with trial representatives (i.e. unvalidated data). This sample included 36 trials reporting, 41 unique intervention arms who shared unpublished intervention materials. Study characteristics are presented in **Supplementary File 5.** Of note this data set includes BCTs coded to ‘unspecified target behaviour’ and ‘maybe’ presence of BCTs.

**Parental target behaviours**

**Table S6:** Frequency of specific target parental behaviours and domains coded in early child obesity prevention interventions^1^ **(unvalidated data)**

| **Target parental behaviour domain and specific target parental behaviours** | **Number of intervention arms (N=41)** |
| --- | --- |
| **Infant (milk) feeding practices** | **31** |
| Promoting and/or sustaining breastfeeding, including exclusive breastfeeding to 6 months of age | 26 |
| Feeding formula appropriately, if necessary (e.g. making formula per package instructions, feeding in response to the infant’s hunger/satiety cues, feeding with suitable types of formula) | 20 |
| Avoiding overfeeding, by not supplementing breastmilk with formula | 6 |
| Delaying introduction of solid foods (complementary feeding) until 6 months of age | 24 |
| **Food provision and parent feeding practices** | **36** |
| *Behaviours related to dietary intake* |  |
| Providing appropriate types of foods (e.g. vegetables, meat and alternatives, fruits, whole grains, dairy) | 32 |
| Providing age-appropriate portions of each food group (i.e. portion sizes; incl. limiting portions of milk) | 17 |
| Limiting provision of certain foods and drinks (e.g. energy-dense, nutrient poor foods, sugar-sweetened beverages) | 26 |
| *Behaviours related to feeding practices* |  |
| Offering foods repeatedly that have previously been rejected | 15 |
| Offering foods and drinks in response to infants’ hunger/satiety cues (e.g. letting the infant decide how much they eat, not pressuring to eat) | 23 |
| Avoiding use of food to control (or reward) the infant’s emotions, behaviour or consumption of other foods | 10 |
| Providing regular meal routines (incl. eating together, limiting distractions) | 16 |
| **Movement practices** | **23** |
| *Behaviours related to physical activity* |  |
| Placing infant on their stomach for prone play (‘tummy time’) | 16 |
| Promoting age-appropriate physical activity such as active play, outdoor play, activities relating to fundamental movement skills | 20 |
| Providing toys that promote movement such as balls and toys on wheels | 5 |
| *Behaviours related to sedentary behaviour* |  |
| Limiting the amount of time the infant is restrained (e.g. prams/strollers, high chairs, strapped on a caregivers back) | 8 |
| Limiting the amount of time the infant is exposed to screens (e.g. television, mobile devices) | 15 |
| Providing alternatives to screen time | 3 |
| **Sleep health practices** | **12** |
| Promoting regular sleep routine (e.g. calm, quiet, soothing) | 11 |
| Letting the infant settle back to sleep when stirring/crying during sleep cycle (e.g. leaving the room, only picking up infant when awake) | 2 |
| Promoting a positive sleep environment (e.g. quiet, darkened, warm) | 7 |
| Placing infant in cot/bassinet while awake and letting infant learn to fall asleep (e.g. following infant’s signs of tiredness) | 5 |
| Avoiding bed-sharing / co-sleeping (i.e. sleeping with the infant in the same bed) | 3 |
| Maximising day-night differences (e.g. lights on and play in the day, lights off and sleep at night) | 6 |

^1^ N=36 trials, reporting N=41 unique intervention arms that completed the validation process; interventions could target one or more behaviours

**Table S7:** Frequency of combinations of target parental behaviour domains coded in early child obesity prevention interventions **(unvalidated data)**

| **Combination of target behaviour domains** | **Number of intervention arms (N=41)** |
| --- | --- |
| Infant feeding practices, Food provision and parent feeding practices, and Movement practices | 11 |
| All domains | 8 |
| Infant feeding practices, and Food provision and parent feeding practices | 7 |
| Food provision and parent feeding practices | 5 |
| Infant feeding practices | 3 |
| Food provision and parent feeding practices, and Movement practices | 3 |
| Sleep health practices | 1 |
| Infant feeding practices, and Sleep health practices | 1 |
| Infant feeding practices, Food provision and parent feeding practices, and Sleep health practices | 1 |
| Food provision and parent feeding practices, Movement practices, and Sleep health practices | 1 |
| Movement practices | 0 |
| Infant feeding practices, and Movement practices | 0 |
| Food provision and parent feeding practices, and Sleep health practices | 0 |
| Movement practices, and Sleep health practices | 0 |
| Infant feeding practices, Movement practices, and Sleep health practices | 0 |

**Delivery features**

**Table S8** presents delivery features used. Studies ranged from no underpinning theory (n=11) to being informed by multiple behaviour change theories, theories and frameworks for intervention design and delivery, or intervention design processes (2: n=9, 3: n=7, 4: n=2, 5: n=1, 7: n=1).

**Table S8:** Summary of the most common delivery features used in early child obesity prevention interventions **(unvalidated data)**

| **Delivery features**^a^ | **Categories** | **Number of intervention arms (n=41)** |
| --- | --- | --- |
| **Why – theory: Rational, theory or goal** | **Behaviour change theory** |  |
|  | Social Cognitive Theory | 14 |
|  | Health Belief Model | 6 |
|  | Trans Theoretical Model | 3 |
|  | Social Learning Theory | 2 |
|  | I-change model | 1 |
|  | Theory of planned behaviour | 3 |
|  | **Theories, models and frameworks for intervention content** |  |
|  | Anticipatory guidance | 9 |
|  | Health self-empowerment theory | 3 |
|  | Intervention mapping | 3 |
|  | Parenting support theory | 3 |
|  | Responsive parenting framework | 5 |
|  | Theoretical Domains Framework | 2 |
|  | Motivational interviewing | 2 |
|  | Attachment theory | 2 |
|  | Family partnership model | 1 |
|  | Health Empowerment-Focused Health-Smart  Behaviour Program Institute of Medicine  guidelines | 1 |
|  | Health literacy principles | 1 |
|  | Model of planned promotion | 1 |
|  | Social influence theory | 1 |
|  | Social Network Theory | 1 |
|  | Solution-focus Support | 1 |
|  | The Elaboration Likelihood Method | 1 |
|  | The Precaution Adoption Process Model | 1 |
|  | The Theory of Reasoned Action | 1 |
|  | The UK Medical Research Council Framework | 1 |
|  | Theories of Information Processing | 1 |
|  | Modify Parental Behaviours | 1 |
|  | Implementation intention | 1 |
|  | **Intervention development process** |  |
|  | Intervention Mapping | 3 |
|  | Behaviour change wheel | 1 |
|  | Model of planned promotion | 1 |
|  |  |  |
|  | No theory used (regardless of type) | 11 |
| **What – materials:** Physical or | Written materials | 33 |
| informational materials, including | DVD / video | 18 |
| provided to participants | Newsletters | 2 |
|  | Website | 4 |
|  | Mobile application | 4 |
|  | Tangible tools | 1 |
|  | Other (e.g. social media group, magnets) | 13 |
| **What – procedures:** Procedures, | Didactic sessions | 40 |
| activities, processed used in the | Peer/ facilitator support | 25 |
| intervention | Interactive activities | 9 |
|  | Group discussion | 13 |
| **Who provided – intervention delivered** | **Health professional** | **29** |
| **by^b^:** Expertise, background | Medical doctor | 7 |
|  | Generalist medical practitioner | 0 |
|  | Specialist medical practitioner | 7 |
|  | Nursing and midwifery professional | 15 |
|  | Nursing professional | 9 |
|  | Midwifery professional | 0 |
|  | Other health professional | 14 |
|  | Physiotherapist | 1 |
|  | Dietitian and nutritionist | 10 |
|  | Health professionals not elsewhere classified | 3 |
|  | **Teaching professional** | **2** |
|  | Other teaching professional | 2 |
|  | Teaching professionals nor elsewhere classified | 1 |
|  | **Legal, social and cultural professional** | **3** |
|  | Social and religious professional | 3 |
|  | Psychologist | 3 |
|  | Social work and counselling professional | 0 |
|  | **Health associate professional** | **6** |
|  | Nursing and midwifery associate professional | 0 |
|  | Nursing associate professional | 0 |
|  | Other health associate professional | 6 |
|  | Community health worker | 5 |
|  | Medical assistant | 1 |
|  | **Legal, social, cultural and related associate professional** | **2** |
|  | Sport and fitness worker | 2 |
|  | **Personal care worker** | **2** |
|  | Child care worker and teachers aide | 2 |
|  | Child care worker | 2 |
|  | **Researcher not otherwise specified** | **2** |
|  | **Higher education university student** | **3** |
|  | Not applicable | 0 |
|  |  |  |
| **Training for the intervention:** | Yes | 27 |
|  | No | 0 |
|  | Not applicable | 0 |
| **How**  **– delivery mode^c^**: | **Human interactional** | **34** |
|  | Face to face | 33 |
|  | At-a-distance | 0 |
|  | **Printed material** | **32** |
|  | Letter | 1 |
|  | Public notice | 1 |
|  | Printed publication | 30 |
|  | Labelling | 1 |
|  | **Electronic** | **29** |
|  | Call | 16 |
|  | Audio call | 8 |
|  | Video call | 2 |
|  | Messaging | 8 |
|  | Playable electronic storage | 11 |
|  | Mobile application | 5 |
|  | Email | 3 |
|  | Website | 4 |
| **Delivery mode combinations** | Human interaction only | 1 |
|  | Printed material only | 1 |
|  | Electronic only | 4 |
|  | Human interaction + Printed material | 10 |
|  | Human interaction + Electronic | 4 |
|  | Printed material + Electronic | 2 |
|  | Human interaction + Printed material + Electronic | 19 |
|  |  |  |
| **Individual / Group:** | Individual (i.e., one-on-one) | 33 |
|  | Group | 17 |
|  |  |  |
| **Unidirectional / Interactional** | Unidirectional | 15 |
|  | Interactional | 26 |
|  |  |  |
| **Synchronous / Asynchronous** | Synchronous | 35 |
|  | Asynchronous | 8 |
| **Where – intervention setting^d^**: Location | **Residential facility** | **25** |
|  | Household residence**^e^** | 25 |
|  | **Healthcare facility** | **13** |
|  | Hospital facility | 2 |
|  | Hospital outpatient clinic facility | 0 |
|  | Doctor-led primary care facility | 2 |
|  | Community healthcare facility | 8 |
|  | Community outpatient clinic facility | 2 |
|  | **Educational facility** | **6** |
|  | Early year facility | 2 |
|  | University facility | 4 |
|  | **Community facility** | **3** |
|  | Library facility | 1 |
|  | **Research facility** | **1** |
| **Intervention setting combinations** | Residential facility only | 18 |
|  | Healthcare facility only | 9 |
|  | Education facility only | 4 |
|  | Community facility only | 2 |
|  | Residential + Healthcare facilities | 4 |
|  | Residential + Education facilities | 2 |
|  | Residential + research facilities | 1 |
|  | Healthcare + Community facilities | 1 |
| **When and how much – intervention** | Total intervention duration in weeks *(median, range)* | 46.85 (0.3, 172.5) |
| **dose^f^**: | Total number of contacts *(median, range)* | 8 (1, 105) |
|  | Frequency of contact: |  |
|  | < Weekly | 2 |
|  | Weekly to < monthly | 13 |
|  | Monthly or greater | 26 |
|  | Average duration of contact in hours (*median, range)* | 8 (0.5, 30) |
| **Tailoring: If the intervention was planned to be personalised, titrated or** | Yes – included element of tailoring | 21 |
| **adapted at the participant level** | No | 1 |
| **Modifications:** If the intervention was | Yes – intervention modified | 4 |
| modified during the study at the intervention level | No | 1 |
| **Fidelity:** Planned and/or Actual | Yes | 32 |
|  | No | 9 |

^a^ Adapted from Hoffmann et al.^36^ An intervention arm could be coded to multiple categories within a delivery feature cluster. One intervention arm in the primary analysis arm did not complete validation process for delivery features.

^b^ Intervention Source Ontology v3 <https://osf.io/zfn25/>

^c^ Mode of Delivery Ontology <https://osf.io/4j2xh/>

^d^ Intervention Setting Ontology v1 <https://osf.io/g8qfv/>

^e^ Household residence was assumed to be where participants accessed digital/remote intervention content.

^f^ Intervention duration reported in months were multiplied by 4.3 to estimate duration in weeks, (n=1 missing data). Number, frequency and duration of contact for intervention delivery excluded data collection contact time (n=2 missing data, where contact was variable and unable to be estimated).

**Behaviour Change Techniques**

**Table S9** presents the coded BCTs by target parental behaviour domain. **Table S10** shows the average number of BCTs coded per intervention arm, by target parental behaviour domain.

**Table S9:** Frequency of Behaviour Change Techniques coded in early child obesity prevention interventions (N=41) by target parental behaviour domain^a^ **(unvalidated data)**

| **BCT number and label** | **Infant (milk) feeding practices** | **Food provision and parent feeding practices** | **Movement practices** | **Sleep health practices** | **Unspecified target behaviour** | **Overall**  **(all behaviour domains tallied,** |
| --- | --- | --- | --- | --- | --- | --- |
|  | **(n=34)** | **(n=38)** | **(n=25)** | **(n=17)** | **(n=21)** | **N=135)** |
| **1. Goals and planning** |  |  |  |  |  |  |
| 1.1 Goal setting (behaviour) | 9 | 13 | 11 | 4 | 3 | 40 |
| 1.2 Problem solving | 14 | 15 | 6 | 3 | 3 | 41 |
| 1.3 Goal setting (outcome) | 0 | 0 | 0 | 0 | 0 | 0 |
| 1.4 Action planning | 3 | 5 | 4 | 2 | 1 | 15 |
| 1.5 Review behaviour goal(s) | 6 | 7 | 6 | 2 | 3 | 24 |
| 1.6 Discrepancy between current behaviour and goal | 1 | 1 | 1 | 1 | 0 | 4 |
| 1.7 Review outcome goal(s) | 0 | 0 | 0 | 0 | 0 | 0 |
| 1.8 Behavioural contract | 0 | 0 | 0 | 0 | 0 | 0 |
| 1.9 Commitment | 0 | 0 | 0 | 0 | 0 | 0 |
| **2. Feedback and monitoring** |  |  |  |  |  |  |
| 2.1 Monitoring of behaviour by others without feedback | 1 | 1 | 1 | 0 | 0 | 3 |
| 2.2 Feedback on behaviour | 8 | 6 | 4 | 2 | 0 | 20 |
| 2.3 Self-monitoring of behaviour | 1 | 4 | 2 | 2 | 0 | 9 |
| 2.4 Self-monitoring of outcome(s) of behaviour | 2 | 2 | 0 | 0 | 1 | 5 |
| 2.5 Monitoring outcome(s) of behaviour by others without feedback | 0 | 0 | 0 | 0 | 0 | 0 |
| 2.6 Biofeedback | 0 | 0 | 0 | 0 | 0 | 0 |
| 2.7 Feedback on outcome(s) of behaviour | 1 | 0 | 0 | 0 | 3 | 4 |
| **3. Social support** |  |  |  |  |  |  |
| 3.1 Social support (unspecified) | 26 | 19 | 15 | 9 | 7 | 76 |
| 3.2 Social support (practical) | 12 | 10 | 12 | 6 | 7 | 47 |
| 3.3 Social support (emotional) | 1 | 1 | 0 | 0 | 1 | 3 |
| **4. Shaping knowledge** |  |  |  |  |  |  |
| 4.1 Instruction on how to perform a behaviour | 32 | 36 | 0 | 0 | 0 | 68 |
| 4.2 Information about antecedents | 0 | 1 | 0 | 0 | 0 | 1 |
| 4.3 Re-attribution | 0 | 0 | 0 | 0 | 0 | 0 |
| 4.4 Behavioural experiments | 1 | 0 | 0 | 0 | 0 | 1 |
| **5. Natural consequences** |  |  |  |  |  |  |
| 5.1 Information about health consequences | 28 | 28 | 22 | 9 | 0 | 87 |
| 5.2 Salience of consequences | 0 | 5 | 1 | 0 | 1 | 7 |
| 5.3 Information about social and environmental consequences | 14 | 10 | 11 | 2 | 0 | 37 |
| 5.4 Monitoring of emotional consequences | 0 | 0 | 0 | 0 | 0 | 0 |
| 5.5 Anticipated regret | 0 | 0 | 0 | 0 | 0 | 0 |
| 5.6 Information about emotional consequences | 0 | 2 | 3 | 0 | 0 | 5 |
| **6. Comparison of behaviour** |  |  |  |  |  |  |
| 6.1 Demonstration of the behaviour | 13 | 21 | 11 | 4 | 1 | 50 |
| 6.2 Social comparison | 3 | 3 | 3 | 3 | 1 | 13 |
| 6.3 Information about others’ approval | 0 | 0 | 0 | 0 | 0 | 0 |
| **7. Associations** |  |  |  |  |  |  |
| 7.1 Prompts / cues | 4 | 14 | 7 | 1 | 1 | 27 |
| 7.2 Cue signalling reward | 0 | 0 | 0 | 0 | 0 | 0 |
| 7.3 Reduce prompts/cues | 0 | 0 | 0 | 0 | 0 | 0 |
| 7.4 Remove access to the reward | 0 | 0 | 0 | 0 | 0 | 0 |
| 7.5 Remove aversive stimulus | 0 | 0 | 0 | 0 | 0 | 0 |
| 7.6 Satiation | 0 | 0 | 0 | 0 | 0 | 0 |
| 7.7 Exposure | 0 | 0 | 0 | 0 | 0 | 0 |
| 7.8 Associative learning | 0 | 0 | 0 | 0 | 0 | 0 |
| **8. Repetition and substitution** |  |  |  |  |  |  |
| 8.1 Behavioural practice / rehearsal | 17 | 33 | 22 | 9 | 0 | 81 |
| 8.2 Behavioural substitution | 11 | 16 | 12 | 4 | 0 | 43 |
| 8.3 Habit formation | 0 | 5 | 5 | 6 | 0 | 16 |
| 8.4 Habit reversal | 0 | 1 | 1 | 1 | 0 | 3 |
| 8.5 Overcorrection | 0 | 0 | 0 | 0 | 0 | 0 |
| 8.6 Generalisation of a target behaviour | 0 | 1 | 1 | 1 | 0 | 3 |
| 8.7 Graded tasks | 2 | 2 | 2 | 2 | 0 | 8 |
| **9. Comparison of outcomes** |  |  |  |  |  |  |
| 9.1 Credible source | 22 | 22 | 17 | 7 | 1 | 69 |
| 9.2 Pros and cons | 7 | 0 | 1 | 0 | 0 | 8 |
| 9.3 Comparative imagining of future outcomes | 0 | 0 | 0 | 2 | 0 | 2 |
| **10. Reward and threat** |  |  |  |  |  |  |
| 10.1 Material incentive (behaviour) | 0 | 0 | 0 | 0 | 0 | 0 |
| 10.2 Material reward (behaviour) | 0 | 0 | 0 | 0 | 0 | 0 |
| 10.3 Non-specific reward | 1 | 1 | 1 | 1 | 1 | 5 |
| 10.4 Social reward | 11 | 8 | 9 | 3 | 2 | 33 |
| 10.5 Social incentive | 0 | 0 | 0 | 0 | 0 | 0 |
| 10.6 Non-specific incentive | 0 | 0 | 0 | 0 | 0 | 0 |
| 10.7 Self-incentive | 0 | 0 | 0 | 0 | 0 | 0 |
| 10.8 Incentive (outcome) | 0 | 0 | 0 | 0 | 0 | 0 |
| 10.9 Self-reward | 2 | 1 | 1 | 1 | 1 | 6 |
| 10.10 Reward (outcome) | 0 | 0 | 0 | 0 | 0 | 0 |
| 10.11 Future punishment | 0 | 0 | 0 | 0 | 0 | 0 |
| **11. Regulation** |  |  |  |  |  |  |
| 11.1 Pharmacological support | 0 | 0 | 0 | 0 | 0 | 0 |
| 11.2 Reduce negative emotions | 6 | 10 | 2 | 3 | 7 | 28 |
| 11.3 Conserving mental resources | 0 | 1 | 0 | 0 | 0 | 1 |
| 11.4 Paradoxical instructions | 0 | 0 | 0 | 0 | 0 | 0 |
| **12. Antecedents** |  |  |  |  |  |  |
| 12.1 Restructuring the physical environment | 1 | 13 | 11 | 2 | 0 | 27 |
| 12.2 Restructuring the social environment | 3 | 11 | 9 | 3 | 1 | 27 |
| 12.3 Avoidance/ reducing exposure to cues for the behaviour | 0 | 1 | 0 | 0 | 0 | 1 |
| 12.4 Distraction | 0 | 0 | 0 | 0 | 0 | 0 |
| 12.5 Adding objects to the environment | 3 | 12 | 8 | 3 | 2 | 28 |
| 12.6 Body changes | 0 | 0 | 0 | 0 | 0 | 0 |
| **13. Identity** |  |  |  |  |  |  |
| 13.1 Identification of self as role model | 0 | 23 | 18 | 0 | 1 | 42 |
| 13.2 Framing / reframing | 1 | 1 | 1 | 1 | 1 | 5 |
| 13.3 Incompatible beliefs | 0 | 0 | 0 | 0 | 0 | 0 |
| 13.4 Valued self-identity | 0 | 0 | 0 | 0 | 0 | 0 |
| 13.5 Identity associated with changed behaviour | 0 | 0 | 0 | 0 | 0 | 0 |
| **14. Scheduled consequences** |  |  |  |  |  |  |
| 14.1 Behaviour cost | 0 | 0 | 0 | 0 | 0 | 0 |
| 14.2 Punishment | 0 | 0 | 0 | 0 | 0 | 0 |
| 14.3 Remove reward | 0 | 0 | 0 | 0 | 0 | 0 |
| 14.4 Reward approximation | 0 | 0 | 0 | 0 | 0 | 0 |
| 14.5 Rewarding completion | 0 | 0 | 0 | 0 | 0 | 0 |
| 14.6 Situation-specific reward | 0 | 0 | 0 | 0 | 0 | 0 |
| 14.7 Reward incompatible behaviour | 0 | 0 | 0 | 0 | 0 | 0 |
| 14.8 Reward alternative behaviour | 0 | 0 | 0 | 0 | 0 | 0 |
| 14.9 Reduce reward frequency | 0 | 0 | 0 | 0 | 0 | 0 |
| 14.10 Remove punishment | 0 | 0 | 0 | 0 | 0 | 0 |
| **15. Self-belief** |  |  |  |  |  |  |
| 15.1 Verbal persuasion about capability | 2 | 3 | 3 | 2 | 1 | 11 |
| 15.2 Mental rehearsal of successful performance | 0 | 0 | 1 | 0 | 1 | 2 |
| 15.3 Focus on past success | 1 | 1 | 1 | 1 | 0 | 4 |
| 15.4 Self-talk | 0 | 0 | 1 | 0 | 0 | 1 |
| **16. Covert learning** |  |  |  |  |  |  |
| 16.1 Imaginary punishment | 0 | 0 | 0 | 0 | 0 | 0 |
| 16.2 Imaginary reward | 0 | 0 | 0 | 0 | 0 | 0 |
| 16.3 Vicarious consequences | 0 | 0 | 0 | 0 | 0 | 0 |

**Table S10:** Number of total Behaviour Change Techniques per intervention arm, and target parental behaviour domain **(unvalidated data)**

|  | **Infant (milk) feeding practices** | **Food provision and parent feeding practices** | **Movement practices** | **Sleep health practices** |
| --- | --- | --- | --- | --- |
|  | **Median (Range)** | **Median (Range)** | **Median (Range)** | **Median (Range)** |
| Number of total BCTs per intervention arm | 11 (2, 29) | 13 (3, 32) | 13 (2, 29) | 7 (2, 21) |
